# Antibacterial and Antioxidant Efficacies of Secondary Metabolites from the Roots of *Cyphostemma adenocaule*: A Combined *In Vitro* and *In Silico* Study

**DOI:** 10.1155/2024/1679695

**Published:** 2024-03-06

**Authors:** Hadush Gebrehiwot, Yadessa Melaku, Muhdin Aliye, Urgessa Ensermu, Aman Dekebo, Milkyas Endale, Daniel Rentsch, Mo Hunsen

**Affiliations:** ^1^Department of Applied Chemistry, Adama Science and Technology University, P.O. Box 1888, Adama, Ethiopia; ^2^Department of Applied Biology, Adama Science and Technology University, P.O. Box 1888, Adama, Ethiopia; ^3^Institute of Pharmaceutical Sciences, Adama Science and Technology University, P.O. Box 1888, Adama, Ethiopia; ^4^Traditional and Modern Medicine Research and Development Directorate, Armauer Hansen Research Institute, P.O. Box 1242, Addis Ababa, Ethiopia; ^5^Laboratory for Functional Polymers, Empa, Swiss Federal Laboratories for Materials Science and Technology, Überlandstrasse 129, Dübendorf 8600, Switzerland; ^6^Department of Chemistry, Kenyon College, Gambier, OH 43022, USA

## Abstract

*Cyphostemma adenocaule* is a therapeutic plant traditionally used to treat rabies, snake bite, diarrhea, and wound healing. To address the bioactive compounds exhibiting these activities, we performed a comprehensive study on the roots of the plant. Thus, the present study aims to inspect the *in vitro* antioxidant and antibacterial efficacies of compounds isolated from the combined dichloromethane : methanol (1 : 1) and methanol extracts of *C. adenocaule* along with the *in silico* study of their interaction with selected protein targets. The silica gel column chromatography technique was used for the isolation of compounds, and the antibacterial and antioxidant activities were evaluated using agar disc diffusion and DPPH radical scavenging assays, respectively. Furthermore, *in silico* molecular docking screening, pharmacokinetics, and toxicity protocols of the compound isolates were performed to offer the potential applications of the compounds in developing novel medications. A BIOVIA Discovery Studio in combination with AutoDock Vina 4.2 software, SwissADME, and ProTox-II prediction web tools were used to generate the molecular docking, pharmacokinetics, and toxicity profiles, respectively. Notably, the chromatographic separation of the combined extracts yielded six known compounds, namely, *β-*sitosterol (**1**), 3-hydroxyisoagatholactone (**2**), *ε*-viniferin (**3**), myricetin (**4**), tricuspidatol A (**5**), and parthenocissin A (**6**). The *in vitro* antibacterial activities revealed the highest inhibition zone by tricuspidatol A (**5**) (16.67 ± 0.47), showcasing its potent activity against *S. aureus* at 2 mg/mL, compared to ciprofloxacin (21.50 ± 0.41). *ε*-Viniferin (**3**) (IC_50_: 0.32 *μ*g/mL) exhibited greater antioxidant activity than the others and displayed promising results compared to ascorbic acid (0.075 *μ*g/mL). The molecular docking study revealed the highest binding affinity by *ε-*viniferin (**3**) (−9.9 kcal/mol) against topoisomerase II *α.* 3-Hydroxyisoagatholactone (**2**) and *ε*-viniferin (**3**) fulfilled Lipinski's rule with no violation, and the organ toxicity predictions revealed that all the compounds showed no cytotoxicity and hepatotoxicity effects. Thus, this study's combined *in vitro* and *in silico* outcomes suggest the potential use of the isolated compounds in drug discovery and support the traditional relevance of *C. adenocaule*.

## 1. Introduction

The biological activities and therapeutic values of medicinal plants rely on the existence of phytocompounds which generate a certain physiological response in the human body [1]. The most vital of these biologically active substances includes terpenoids, alkaloids, saponins, polyphenolic compounds, tannins, essential oils, and vitamins [2]. Plenty of studies revealed that these bioactive compounds exhibit a broad array of biological properties such as anticancer, antioxidant, anti-inflammatory, antifungal, antibacterial, antidiabetic, and antimalarial activities [3].

**Figure 1:**
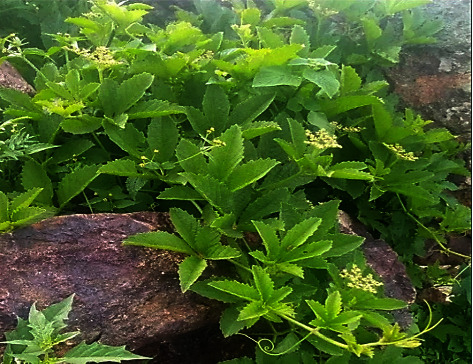
*Cyphostemma adenocaule* (picture by Hadush G., October 2021, Adama, Ethiopia).

The genus *Cyphostemma* (Vitaceae family) contains more than 250 taxonomically identified species disseminated all over the tropics and subtropics of the world [4] with a wide spectrum of traditional uses to cure toothache, rabies, snake bite, tumor, inflammation, malaria, marasmus, kwashiorkor in children, diarrhea, syphilis, and urinary and tract infections [5, 6]. *Cyphostemma adenocaule* (Steud. ex A. Rich.) (Figure 1) is one of the most prevalent vegetable plants grown in many parts of Africa, i.e., Ethiopia, Angola, Nigeria, Eritrea, Ghana, Uganda, Congo, Senegal, Malawi, and Mozambique [7, 8]. In Ethiopia, the plant has different vernacular names, such as *Hareg Temen* (Tigrinya), *Aserkuh Aserkush* (Amharic), and *Hida Bofa* (Afan Oromo) [9]. In Tigray, Northern Ethiopia, the fresh roots of the plant have long been used against rabies, snake bite, and wound healing [10] and traditional healers around Gondar, Ethiopia, use the boiled root of *C. adenocaule* with milk and drink it to treat rabies [11].

The phytochemistry of the plant revealed many natural substances including flavonoids, stilbenes derivatives, coumarins, alkaloids, terpenoids, saponins, and tannins [12, 13]. These phytocompounds reveal diverse biological activities, including antifungal, antioxidant, antibacterial, chemopreventive, hepatoprotective, and antiproliferative activities [14, 15]. *β*-Sitosterol, betulin, betulinic acid, lupeol, cyphostemmic acids (A, B, C, and D), zizyberanal acid, and epigouanic acid A were among the reported terpenoid compounds in the stem barks and roots extract of *C. adenocaule* [13, 16]. Thus, motivated by its wide spectrum of traditional uses, the present study aims to inspect the *in vitro* antioxidant and antibacterial efficacies and *in silico* cytotoxic properties of compounds isolated from the combined CH_2_Cl_2_ : MeOH (1 : 1) and methanol root extracts of *C. adenocaule* along with computational study of their interaction with selected protein targets.

## 2. Materials and Methods

### 2.1. Plant Material

The roots of *C. adenocaule* (wild climbing herb) were harvested from the mountains of Adama City, Ethiopia, in October 2021. After collection, the plant material was authenticated by Mr. Melaku Wendafrash (chief botanist). A voucher number of the specimen was designated by an accession number (HCA003) and deposited at the National Herbarium of Ethiopia, Department of Biology, Addis Ababa University, Addis Ababa, Ethiopia. The plant samples were washed successively with tap and distilled water and subjected to air drying for three weeks at room temperature without direct exposure to sunlight.

### 2.2. General Experimental Procedures

The roots of *C. adenocaule* were finely milled with a grinder (Shanghai Jingke, JK-HSG-100A, China) and extracted with CH_2_Cl_2_ (98% Alpha Chemika, India) : MeOH (1 : 1) and MeOH (99.8%, Loba Chemie, India). The solvents were freed using a rotary evaporator (DW-RE-3000, China) maintaining the temperature at 40°C. The crude extracts were chromatographed on silica gel (60–120 and 60–200 mesh, Merck, India) using column chromatography and eluted by dissolving in an appropriate solvent. For purity analysis, TLC aluminum plates (0.25 mm) coated with high-purity grade silica gel (230–400 mesh, pore size 60 Å, Merck Grade 64271, Darmstadt, Germany) were applied. The fractions of compounds were visualized under a UV light (UV cabinet, UV4AC6/2, CBIO Bioscience and Technologies, Beijing, China, at 254 nm and 365 nm), spraying with 1% vanillin-sulfuric acid reagent followed by direct heating over a stove at 110°C. NMR spectral results were recorded in deuterated chloroform and methanol on 400 MHz Bruker AVANCE III NMR instruments. The chemical shift (*δ*) values were conveyed in ppm and referred against the resonance values of the solvents at 3.31 ppm (*δ*_*H*_) and 49.1 ppm (*δ*_*C*_) for deuterated methanol and 7.28 ppm (*δ*_*H*_) and 77.2 ppm (*δ*_*C*_) for deuterated chloroform. Melting points were determined on a Japson Melting Point Apparatus (JA90161, India). Furthermore, the antioxidant properties of the plant extracts were achieved on a UV-Vis spectrophotometer (CE4001 UV/VIS, Cambridge, UK) equipped with tungsten and deuterium lamps. A hood with UV-radiation and laminar air flow, dimethyl sulfoxide, Muller–Hinton Agar (MHA) (Micro Express, India), sterilized needle, autoclave, incubator, micropipette (1000 *μ*L), and Petri dishes (90 mm) were used for the antibacterial testing assays. All the solvents and reagents used were of analytical grade.

### 2.3. Extraction and Isolation

The pulverized roots of *C. adenocaule* (600 g) were successively extracted by a cold maceration technique with 3 L of CH_2_Cl_2_ : MeOH (1 : 1) followed by an equal volume of methanol for 72 h each with continuous shaking over an orbital shaker (VRN-200, Gemmy industries, Taiwan) at room temperature. The extracts were filtered over gravity with a Whatman No. 1 filter paper, and the filtrates were concentrated *in vacuo* on a rotary evaporator at 40°C [17, 18] to generate 22 and 28 g of CH_2_Cl_2_ : MeOH (1 : 1) and methanol extracts, respectively. Both extracts exhibited similar TLC profiles and were mixed to obtain 50 g of the crude extract.

The combined crude extracts (20 g) were subjected to silica gel column chromatographic separation (180 g silica gel, 60–120 mesh as stationary phase) and eluted with increasing gradient of *n*-hexane : ethyl acetate to chloroform : methanol mixtures successively. A total of 165 fractions (50 mL each) were collected, and their purities were monitored using TLC and UV lamp at 254 and 365 nm, respectively. Finally, the pure compounds were characterized using spectroscopic techniques (1D and 2D NMR) [19]. The fractionation process of the extract failed to generate pure compounds; therefore, further purifications were performed for fractions with promising TLC profiles. Accordingly, fractions 10–15 (98.5 mg), collected with 30% EtOAc in *n*-hexane, were purified using the gradient elution (34 g silica gel) of 100% *n*-hexane up to 50% EtOAc in *n*-hexane to yield *β-*sitosterol (**1**) (23.8 mg, *R*_*f*_: 0.48 in 20% EtOAc in *n*-hexane). Fractions 21–23 (116.2 mg), collected with 50% EtOAc in *n*-hexane, were mixed and purified using the gradient elution (33 g silica gel) of 20–70% EtOAc in *n*-hexane to yield 3-hydroxy isoagatholactone (**2**) (12.9 mg, *R*_*f*_: 0.68 in 40% EtOAc in *n*-hexane). Fractions 30–33 (150.4 mg), collected with 75% EtOAc in *n*-hexane, were mixed and purified with PTLC (mobile phase: 40% EtOAc in *n*-hexane) to yield *ε*-viniferin (**3**) (32.0 mg, *R*_*f*_: 0.67 in 65% EtOAc in *n*-hexane). The PTLC purification (mobile phase: 40% EtOAc in *n*-hexane) of fraction 34 (63.0 mg) isolated using 75% EtOAc in *n*-hexane yielded myricetin (**4**) (19.2 mg, *R*_*f*_: 0.75 in 70% EtOAc in *n*-hexane). Fraction 39 (122.0 mg), isolated with 85% EtOAc in *n*-hexane, was subjected to an isocratic elution (34 g silica gel) with 80% EtOAc in *n*-hexane to yield tricuspidatol A (**5**) (51.2 mg, *R*_*f*_: 0.46 in 70% EtOAc in *n*-hexane). Finally, the PTLC purification (mobile phase: 60% EtOAc in *n*-hexane) of fractions 40–42 (61.0 mg) collected with 90% EtOAc in *n*-hexane as eluent yielded parthenocissin A (**6**) (15.1 mg, *R*_*f*_: 0.58 in 80% EtOAc in *n*-hexane).

### 2.4. *In Vitro* Antibacterial Activity

#### 2.4.1. Bacterial Strains

The isolated compounds were examined *in vitro* for their antibacterial efficiency against four bacterial strains named *Escherichia coli* (ATCC-25922), *Staphylococcus aureus* (ATCC-25923), *Pseudomonas aeruginosa* (ATCC-27853), and *Streptococcus pyogenes* (ATCC-19615) which were selected based on their prevalence and common health problems in Ethiopia, availability, phenotype and genotype information, and relatedness to the ethnomedicinal relevance of the plant. The bacterial pathogens were obtained from the Ethiopian Public Health Institute (EPHI), and the experimental tests were attained at the Research Laboratory of Microbiology, Adama Science and Technology University, Adama, Ethiopia.

#### 2.4.2. Antibacterial Activity Assay

The *in vitro* antibacterial activities were adopted as per the previous experimental protocol [20] using DMSO and ciprofloxacin as negative and positive controls, respectively. The entire tests were performed in triplicate, and the results were reported as mean ± SEM with the help of statistical software.

### 2.5. *In Vitro* Antioxidant Activity

The *in vitro* antioxidant activities were also adopted as per the previous experimental protocol [21] using ascorbic acid as positive control. The absorbance value of a DPPH solution in methanol (negative control) was measured and the antioxidant power of each compound was described in terms of scavenging strength (%) using equation (1) [21]. The results were reported as mean ± SEM and the IC_50_ values were computed from the relationship curve of plots of the concentration of samples against the antioxidant activity mean (%).

(1) $$\begin{array}{c} \text{DPPH radical scavenging power }\left(\%\right)=\left(1-\frac{A}{A_{0}}\right)*100, \end{array}$$

where *A* and *A*_0_ indicate the absorbance of the sample solution in methanol + DPPH radical and DPPH radical in methanol, respectively.

### 2.6. Molecular Docking Studies

The compound isolates were docked against protein targets of the bacterial pathogens and human enzymes to predict their activity and binding affinity. The molecular docking calculations include 2D and 3D structural representations of viable docked poses, binding affinity (kcal/mol), hydrogen bonds, and residual amino acid interactions. Protein targets of *E. coli* DNA gyrase B (PDB ID: 6F86), *P. aeruginosa* PqsA (PDB ID: 5OE3), *S. aureus* PK (PDB ID: 3T07), human myeloperoxidase (PDB ID: 1DNU), and topoisomerase II *α* (PDB ID: 4FM9) were selected for the study. The bacterial protein targets were selected based on the respective strains' *in vitro* antibacterial and antioxidant activities, their metabolic importance to the microorganisms, and the similarity of the compound to the cocrystalized ligands in the protein complex. The 3D crystal structures of the target proteins were retrieved in PDB format from the Protein Data Bank (https://www.rcsb.org), whereas the 3D structures of the compounds were attained from PubChem/Zinc databases, converted to PDB files via Open Babel GUI application, and saved in a similar folder with the proteins. The crystal structures of *E. coli* DNA gyrase B, with a resolution of 1.90 Å possessing a single chain cocrystalized with a native ligand 4-(4-bromo-1H-pyrazol-1-yl)-6-[(ethylcarbamoyl)-amino]-N-(pyridine-3-yl)-pyridine-3-carboxamide (C_17_ H_16_ Br N_7_ O_2_), pyruvate kinase (PK) of *S. aureus* in combination with a naturally occurring bisindole alkaloid with a resolution of 3.30 Å possessing four chains (A, B, C, and D), two ligands, and four phosphate groups and N-terminal domain of PqsA in combination with anthraniloyl-AMP, with a resolution of 1.43 Å possessing four chains (A, B, C, and D) with multiple ligands were retrieved from the Protein Data Bank (PDB). Moreover, the crystal structures of human myeloperoxidase with thiocyanate complex, resolution of 1.85 Å possessing four chains (A, B, C, and D) and multiple ligands and human topoisomerase II *α* bound to DNA, resolution of 2.90 Å which possess three chains (A, C, and D), and a single ligand were attained from the PDB database [22–24].

Water molecules and heteroatoms (phosphate, native ligands) were removed in the Discovery Studio Visualizer software; polar hydrogens were added, and proteins were saved in PDB format. Afterwards, Kollman charges and AD4-type atoms were added for the proper optimizations using MGL tools 1.5.7 and the proteins were saved in PDBQT format. Finally, the lowest energy conformations of the ligands were used as an input to run out the molecular docking simulations [25]. Then, the PDB format of the compounds was computed using MGL tools, Gasteiger charges were added, the torsion angle of the ligand was adjusted, and the proteins were saved in PDBQT format. The active sites were established by studying the binding interaction of the receptor and the ligand and the molecular dockings were run on the active sites of the prepared target proteins. The running process was achieved by building a grid box (50 × 50 × 54 Å) with a grid spacing of 0.375 Å and covering the active sites. The docking process was achieved using the AutoDock Vina 4.2 program and all the files saved in the working directory were run via the command prompt. For each ligand scored, nine different conformations were produced. Conformations of the least (stable) binding affinity and root mean square deviation (RMSD) were chosen for evaluating the interactions between the ligands and receptors. Finally, the docking analysis was computed using the BIOVIA Discovery Studio Visualizer 2021 [26].

### 2.7. *In Silico* Pharmacokinetic Analyses

The compound isolates were studied for their drug candidate likelihoods using the pharmacokinetic parameters, following the protocols of Lipinski et al. [27], Daina et al. [28], Banerjee et al. [29], and Behrouz et al. [30].

### 2.8. Spectral and Statistical Data Analyses

MestReNova software (version 14.2) was used for processing (apodization, Fourier transformation, and phase correction) of NMR raw data. The antibacterial and antioxidant data were tabulated in a Microsoft Excel spreadsheet and the values were conveyed as mean ± standard error of the mean (SEM) (antibacterial) and % of scavenging activity (antioxidant). The antibacterial activities were evaluated by comparing the inhibition zones of the compounds with the control drug (ciprofloxacin) and the solvent (DMSO). The IC_50_ scores of the compounds were computed from the relationship curves of the plots and compared to ascorbic acid.

## 3. Results and Discussion

### 3.1. Structure Elucidations

**Figure 2:**
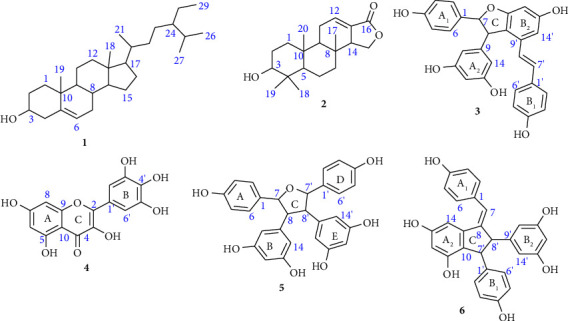
Structural information of the isolated compounds (**1–6**) from *C. adenocaule* (roots).

The silica gel chromatographic separation of the combined CH_2_Cl_2_ : MeOH (1 : 1) and methanol extracts yielded *β-*sitosterol (**1**), 3-hydroxy isoagatholactone (**2**), *ε*-viniferin (**3**), myricetin (**4**), tricuspidatol A (**5**), and parthenocissin A (**6**) (Figure 2). The structural elucidations of the compounds along with their literature references are presented.

Compound **1** (mp; 135–137°C) was isolated as white needles. The measured melting point range was very close to the literature values of *β-*sitosterol (134-135 °C) [31]. Its ^1^H NMR (400 MHz, CDCl_3_) spectrum revealed signals of olefinic proton at *δ*_*H*_ 5.34 (1H, *m,* H-6), suggesting that the compound has a single sp^2^ olefinic proton. The spectrum also showed a signal of oxymethine proton at *δ*_*H*_ 3.51 (1H, *m*) assignable to H-3. The signals at *δ*_*H*_ 2.27 (1H, *m*, H-4a), 2.22 (1H, *m*, H-4b), 1.98 (1H, *m*, H-7a), and 1.94 (1H, *m,* H-7b) suggest diastereotopic protons attributed to the methylene protons adjacent to the sp^2^ unsaturated olefinic carbons. Furthermore, the spectrum exhibited six methyl signals at *δ*_*H*_ 1.00 (3H, *s,* H-19), 0.91 (3H, *d, J* = 6.6 Hz, H-21), 0.83 (3H, *t*, H-29), 0.81 (3H, *d, J* = 6.1 Hz, H-27), 0.80 (3H, *d, J* = 6.2 Hz, H-26), and 0.67 (3H, *s*, H-18). The other proton signals coincided in the range *δ*_*H*_ 1.87−0.90 (25H, *m*) (Supplementary materials, Table S1).

The ^13^C NMR (400 MHz, CDCl_3_) spectrum of the compound exhibited twenty-nine well-resolved carbon signals including two olefinic carbons at *δ*_*C*_ 140.8 (C-5) and 121.8 (C-6) of which the earlier embodies a quaternary carbon, an oxymethine carbon at *δ*_*C*_ 71.9 (C-3), seven sp^3^ methine signals at *δ*_*C*_ 56.8 (C-14), 56.1 (C-17), 50.2 (C-9), 45.9 (C-24), 36.2 (C-20), 32.0 (C-8), and 29.2 (C-25), eleven methylene signals at *δ*_*C*_ 42.4 (C-4), 39.8 (C-12), 37.3 (C-1), 34.0 (C-22), 32.0 (C-7), 31.7 (C-2), 28.3 (C-16), 26.1 (C-23), 24.4 (C-15), 23.1 (C-28), and 21.1 (C-11), six methyl carbons at *δ*_*C*_ 19.9 (C-27), 19.5 (C-19), 19.1 (C-26), 18.8 (C-21), 12.0 (C-18), and 11.9 (C-29), and two sp^3^ quaternary carbons at *δ*_*C*_ 42.3 (C-13) and 36.6 (C-10) (Supplementary materials, Table S1). In addition, the proton NMR assignments were verified by COSY correlations. Thereby, the spectrum showed significant ^3^*J* correlations between H-1b (*δ*_*H*_ 1.07) and H-2a (*δ*_*H*_ 1.81), H-3 (*δ*_*H*_ 3.51) and H-4 (*δ*_*H*_ 2.27, 2.22), H-6 (*δ*_*H*_ 5.34) and H-7 (*δ*_*H*_ 1.98, 1.94), H-20 (*δ*_*H*_ 1.33) and H-21 (*δ*_*H*_ 0.91), H-25 (*δ*_*H*_ 1.64) and H-26, 27 (*δ*_*H*_ 0.80, 81), and H-28 (*δ*_*H*_ 1.20) and H-29 (*δ*_*H*_ 0.83) (Supplementary materials, Table S1). Overall, the spectral data match the reported values of *β-*sitosterol (**1**) [32] (Figure 2).

Compound **2** was isolated as a yellow amorphous substance, and its ^1^H NMR spectrum (400 MHz, CDCl_3_) revealed signals of an olefinic proton at *δ*_*H*_ 6.85 (1H, *dd*, *J* = 3.5, 3.5 Hz, H-12) and oxygenated diastereotopic methylene signals at *δ*_*H*_ 4.36 (1H, *t*, *J* = 9.2 Hz, H-15a) and 4.03 (1H, *t*, *J* = 9.1 Hz, H-15b) together with the ^13^C NMR spectrum produce the structure of a compound associated with a spongiane diterpenoid lactone skeleton. The signals at *δ*_*H*_ 5.28 (1H, brs) and 3.42 (1H, *t*, *J* = 2.9 Hz) suggest hydroxyl (OH) and oxymethine (H-3) protons, respectively. The peaks at *δ*_*H*_ 2.79 (1H, *dd*, *J* = 8.7, 4.4 Hz, H-14) and 2.37 (1H, *dd, J* = 5.6 Hz, H-11a) and 2.32 (1H, *dd, J* *=* 5.7 Hz, H-11b) were assignable to the sp^3^ methine and methylene protons adjacent to the oxymethylene and sp^2^ methine groups, respectively. The spectrum also displayed four groups of methyl protons at *δ*_*H*_ 0.95 (3H, *s*), 0.92 (3H, *s*), 0.85 (3H, *s*), and 0.76 (3H, *s*) assignable to H-20, H-18, H-19, and H-17, respectively (Supplementary materials, Table S2).

In its ^13^C NMR spectrum, compound **2** displayed carbon signals at *δ*_*C*_ 170.3 (C-16), 136.4 (C-12), 127.0 (C-13), and 67.3 (C-15) associated with a spongiane diterpenoid lactone skeleton (also supported by the ^1^H NMR spectral analysis). An additional oxygenated sp^3^ methine signal was observed at *δ*_*C*_ 75.8 assignable to C-3. The spectrum also revealed four methyl signals at *δ*_*C*_ 28.4, 22.2, 15.2, and 14.2 corresponding to C-18, C-19, C-20, and C-17, respectively. Moreover, the carbon signals at *δ*_*C*_ 54.1, 51.1, 49.3, 40.7, 37.5, 37.1, 34.5, 32.8, 25.1, 24.2, and 18.0 attributed to C-5, C-9, C-14, C-7, C-4, C-10, C-8, C-1, C-2, C-11, and C-6, respectively. The types of the carbon signals were verified by DEPT-135 spectrum; thereby, the spectral information revealed one sp^2^ olefinic methine signal at *δ*_*C*_ 136.4 (C-12), two sp^3^ oxymethine and oxymethylene signals at *δ*_*C*_ 75.8 (C-3) and 67.3 (C-15), respectively, three sp^3^ methine carbons, five sp^3^ methylene, and four methyl signals and were consistent with the ^13^C NMR spectral analysis of the compound (Supplementary materials, Table S2).

The spectral analyses of the compound were supported by 2D NMR correlations. Accordingly, the COSY spectrum exhibited ^3^*J* correlations between H-2 (*δ*_*H*_ 2.11, 1.56) and H-3 (*δ*_*H*_ 3.42), H-9 (*δ*_*H*_ 1.43) and H-11 (*δ*_*H*_ 2.37), H-11 (*δ*_*H*_ 2.37) and H-12 (*δ*_*H*_ 6.85), and H-14 (*δ*_*H*_ 2.79) and H-15 (*δ*_*H*_ 4.36, 4.03). In addition, the connectivity was confirmed by HMBC correlations, thereby the spectrum showed ^3^*J* correlations between H-5 (*δ*_*H*_ 1.38) and C-7, 20 (*δ*_*C*_ 40.7, 15.2), H-15 (*δ*_*H*_ 4.36, 4.03), and C-13, 16 (*δ*_*C*_ 127.0, 170.3) (Supplementary materials, Table S2). Finally, the spectral data were in close agreement with the reported values of 3-hydroxyisoagatholactone (**2**) [33] (Figure 2).

Compound **3** was obtained as a pale brown solid and its melting point was determined in the range 180–182 °C (literature mp: 185°C, [34]). Its ^1^H NMR (400 MHz, CD_3_OD) spectrum exhibited signals with a AA′BB′ spin pattern at *δ*_*H*_ 7.14 (2H, *d*, *J* = 8.7 Hz, H-2, 6) and 6.78 (2H, *d*, *J* = 8.7 Hz, H-3, 5) (ring *A*_1_), AA′BB′ spin pattern at *δ*_*H*_ 7.03 (2H, *d*, *J* = 8.8 Hz, H-2′, 6′) and 6.66 (2H, *d*, *J* = 8.8 Hz, H-3′, 5′) (ring *B*_1_), AB spin pattern at *δ*_*H*_ 6.64 (1H, *s*, H-14′) and 6.26 (1H, *s*, H-12′) (ring B_2_), and ABX spin pattern at *δ*_*H*_ 6.21 (1H, *s*, H-12) and 6.18 (2H, *s*, H-10, 14) (ring *A*_2_). The signals appeared at *δ*_*H*_ 6.84 (1H, *d*, *J* = 16.7 Hz) and 6.57 (1H, *d*, *J* = 16.3 Hz) attributed to trans-oriented olefinic protons (H-7′ and H-8′, respectively). The spectrum also displayed signals at *δ*_*H*_ 5.37 (1H, *d*, *J* = 6.6, H-7) and 4.35 (1H, *d*, *J* = 6.6, H-8) corresponding to the oxymethine (H-7) and methine protons (H-8) of the substituted tetrahydrofuran skeleton (ring C) (Supplementary materials, Table S3).

The ^13^C NMR spectrum revealed 22 identifiable signals assignable to 28 carbons. The signals at *δ*_*C*_ 162.7 (C-11′) (ring *B*_2_), 160.0 (C-11, 13) (ring *A*_2_), 159.7 (C-13′) (ring *B*_2_), 158.5 (C-4) (ring *A*_1_), and 158.4 (C-4′) (ring *B*_1_) attributed to the oxygenated carbons of the structure, and other nonoxygenated quaternary carbons appeared at *δ*_*C*_ 147.3 (C-9), 136.8 (C-9′), 133.8 (C-1), 131.2 (C-1′), and 120.0 (C-10′). The spectrum also showed sp^2^ aromatic methine signals at *δ*_*C*_ 128.8 (C-2′, 6′), 128.1 (C-2, 6), 116.4 (C-3, 5), 116.3 (C-3′, 5′), 107.5 (C-10, 14), 104.3 (C-14′), 102.3 (C-12), and 96.9 (C-12′) and sp^2^ olefinic methine signals at *δ*_*C*_ 130.3 (C-7′) and 123.6 (C-8′). The substituted tetrahydrofuran carbons that appeared at *δ*_*C*_ 94.8 and 58.3 were assignable to C-7 and C-8, respectively. Furthermore, the DEPT-135 spectrum indicated eight sp^2^ aromatic methine signals assignable to thirteen carbons, two sp^2^ olefinic methine signals, and two sp^3^ oxymethine and methine carbons which were consistent with the ^13^C NMR spectral data of the compound (Supplementary materials, Table S3).

The connections and substitution patterns of the compound were established from the COSY and HMBC correlations. The COSY spectrum showed correlations between H-2, 6 (*δ*_*H*_ 7.14) and H-3, 5 (*δ*_*H*_ 6.78), H-7 (*δ*_*H*_ 5.37) and H-8 (*δ*_*H*_ 4.35), H-2′, 6′ (*δ*_*H*_ 7.03) and H-3′, 5′ (*δ*_*H*_ 6.66), and H-7′ (*δ*_*H*_ 6.84) and H-8′ (*δ*_*H*_ 6.57) in agreement with rings *A*_1_, *B*_1_ and trans-olefinic protons. The HMBC spectrum also revealed ^3^*J* correlations between H-2/6 (*δ*_*H*_ 7.14) and C-4, 7 (*δ*_*C*_ 158.5, 94.8), ^2^*J* correlations between H-3, 5 (*δ*_*H*_ 6.78) and C-2, 6 (*δ*_*C*_ 128.1), ^3^*J* correlations between H-7 (*δ*_*H*_ 5.37) and C-2/6, 9 (*δ*_*C*_ 128.1 and 147.3, respectively), ^3^*J*, ^2^*J*, ^2^*J,*^3^*J*, ^2^*J*, and ^3^*J* correlations between H-8 (*δ*_*H*_ 4.35) and C-1, 7, 9, 10/14 10′, 11′ (*δ*_*C*_ 133.8, 94.8, 147.3, 107.5, 120.0 and 162.7, respectively), ^3^*J*, ^2^*J*, and ^3^*J* correlations between H-10/14 (*δ*_*H*_ 6.18) and C-8, 11/13, 12 (*δ*_*C*_ 58.3, 160.0 and 102.3, respectively), ^3^*J* correlations between H-2′/6′ (*δ*_*H*_ 7.03) and C-4′, 7′ (*δ*_*C*_ 158.4 and 130.3, respectively), and ^4^*J* correlations between H-7′ (*δ*_*H*_ 6.84) and C-14′ (*δ*_*C*_ 104.3) (Supplementary materials, Table S3). Overall, the spectral analyses were consistent with the literature data of *ε*-viniferin (**3**) [33, 35] (Figure 2).

Compound **4** was collected as yellow crystals, and its melting point was determined in the range of 357–359 °C (literature mp: 357°C, [36]). In its ^1^H NMR (400 MHz, CD_3_OD) spectrum, the compound exhibited signals of aromatic protons at *δ*_*H*_ 7.35 (2H, *s*, H-2′, 6′) with AA′ spin pattern (ring B), 6.38 (1H, *d, J* = 2.0 Hz, H-8) and 6.18 (1H, *d, J* = 2.0 Hz, H-6) with an AB spin pattern (ring A), attributed to a flavonoid skeleton (Supplementary materials, Table S4). The ^13^C NMR spectrum showed a signal at *δ*_*C*_ 177.2 (C-4), suggesting the presence of an *α*, *β*-unsaturated carbonyl compound. The signals at *δ*_*C*_ 165.5 (C-7), 162.4 (C-5), 158.1 (C-9) (ring A), 147.9 (C-2), 137.3 (C-3) (ring C), 146.7 (C-3′, 5′), and 136.9 (C-4′) (ring B) were assignable to the oxygenated quaternary aromatic carbons. The spectrum also revealed nonoxygenated quaternary carbons at *δ*_*C*_ 123.0 (C-1′) and 104.4 (C-10) and sp^2^ aromatic methine signals at *δ*_*C*_ 108.5 (C-2′, 6′), 99.2 (C-6), and 94.3 (C-8) associated with a flavone skeleton. The types of carbon signals were confirmed by DEPT-135 spectral information. Accordingly, the spectrum displayed signals of sp^2^ aromatic methine carbons at *δ*_*C*_ 108.5, 99.2, and 94.3 assignable to (C-2′, 6′), C-6, and C-8, respectively (Supplementary materials, Table S4).

The connections and substitution patterns of the compound were recognized from the HSQC and HMBC correlations. The HSQC spectrum exhibited ^1^*J* correlations between H-6 (*δ*_*H*_ 6.18) and C-6 (*δ*_*C*_ 99.2), H-8 (*δ*_*H*_ 6.38) and C-8 (*δ*_*C*_ 94.3), and H-2′/6′ (*δ*_*H*_ 7.35) and C-2′/6′ (*δ*_*C*_ 108.5). The HMBC spectrum also showed ^3^*J* correlations between H-6 (*δ*_*H*_ 6.18) and C-8, 10 (*δ*_*C*_ 94.3, 104.4), H-8 (*δ*_*H*_ 6.38) and C-6, 10 (*δ*_*C*_ 99.2, 104.4), ^2^*J*, ^3^*J*, ^2^*J*, ^3^*J*, and ^3^*J* correlations between H-2′ (*δ*_*H*_ 7.35) and C-1′, 2, 3′, 4′, 6′ (*δ*_*C*_ 123.0, 147.9, 146.7, 136.9 and 108.5, respectively), and ^2^*J*, ^3^*J*, ^3^*J*, ^3^*J*, and ^2^*J* correlations between H-6′ (*δ*_*H*_ 7.35) and C-1′, 2, 2′, 4′, and 5′ (*δ*_*C*_ 123.0, 147.9, 108.5, 136.9 and 146.7, respectively) (Supplementary materials, Table S4). Finally, the overall data were consistent with the reported values of myricetin (**4**) [37] (Figure 2).

Compound **5** (mp: 165–167 °C) was collected as a grey-brown solid and its melting point agreed with the literature values of tricuspidatol A (mp: 163–169°C) [38]. In its ^1^H NMR (400 MHz, CD_3_OD) spectrum, seven different signals were observed. The doublet signals at *δ*_*H*_ 7.28 (4H, *d, J* = 8.6 Hz) and 6.77 (4H, *d*, *J* = 8.6 Hz) exhibiting an AA′BB′ spin orientation were assignable to the aromatic protons in rings A and D (H-2, 2′, 6, 6′ and H-3, 3′, 5, 5′, respectively). The strong signal at *δ*_*H*_ 6.03 (4H, *s*, H-10, 10′, 14, 14′) and weak signals at *δ*_*H*_ 5.98 (2H, *s*, H-12, 12′) attributed to the aromatic protons with AB_2_ spin orientation in rings B and E. The spectrum also displayed signals of a 2, 3, 4, 5-substituted tetrahydrofuran skeleton at *δ*_*H*_ 5.41 (2H, *d*, *J* = 6.5 Hz) and 3.59 (2H, *d*, *J* = 6.7 Hz) assignable to the oxymethine and methine protons in ring C (H-7, 7′ and H-8, 8′, respectively) (Supplementary materials, Table S5).

The ^13^C NMR spectrum of the compound exhibited ten intense and well-resolved signals, suggesting that the compound affords molecular symmetry. Thus, the signals at *δ*_*C*_ 158.7 (C-11, 11′, 13, 13′) (rings B and E) and 157.9 (C-4, 4′) (rings A and D) were assignable to the oxygenated aromatic carbons, while the signals at *δ*_*C*_ 142.1 (C-9, 9′) (rings B and E) and 133.8 (C-1, 1′) (rings A and D) attributed to the nonoxygenated quaternary carbons of the structure. The spectrum also displayed sp^2^ aromatic methine signals at *δ*_*C*_ 128.8, 116.0, 108.9, and 101.8 corresponding to (C-2, 2′, 6, 6′), (C-3, 3′, 5, 5′), (C-10, 10′, 14, 14′), and (C-12, 12′), respectively. The spectrum also revealed signals at *δ*_*C*_ 85.9 (C-7/7′) and 59.0 (C-8/8′) assignable to the sp^3^ oxymethine and methine carbons of the substituted tetrahydrofuran skeleton, respectively (ring C). Furthermore, the DEPT-135 spectrum showed four sp^2^ aromatic methine signals, one oxymethine, and another one sp^3^ methine carbons, and was consistent with the ^13^C NMR spectral analysis of the compound (Supplementary materials, Table S5).

The connectivity and substitution patterns of the compound were proven by the COSY and HMBC correlations. Accordingly, the COSY spectrum revealed ^3^*J* correlations between H-2/2′/6/6′ (*δ*_*H*_ 7.28) and H-3/3/5/5′ (*δ*_*H*_ 6.77), H-7/7′ (*δ*_*H*_ 5.41), and H-8/8′ (*δ*_*H*_ 3.59). The HMBC spectrum also revealed ^3^*J* correlations between H-2/2′ (*δ*_*H*_ 7.28) and C-4/4′, 6/6′, and 7/7′ (*δ*_*C*_ 157.9, 128.8, and 85.9, respectively), ^3^*J* and ^2^*J* correlations between H-3/3′, 5/5′ (*δ*_*H*_ 6.77) and C-1/1′, 4/4′ (*δ*_*C*_ 133.8 and 157.9, respectively), ^3^*J* and ^2^*J* correlations between H-5/5′ (*δ*_*H*_ 6.77) and C-1/1′, 4/4′ (*δ*_*C*_ 133.8 and 157.9, respectively), ^3^*J* correlations between H-10/10′ (*δ*_*H*_ 6.03) and C-8/8′, 12/12′ (*δ*_*C*_ 59.0 and 101.8, respectively), and ^3^*J* correlations between H-8/8′ (*δ*_*H*_ 3.59) and C-10/10′, 14/14′ (*δ*_*C*_ 108.9) (Supplementary materials, Table S5). After all, the data generated were closely related to the reported values of tricuspidatol A (**5**) [33, 38] (Figure 2).

Compound **6** was isolated as a brown amorphous solid using 90% EtOAc in *n*-hexane as eluent. The ^1^H NMR (400 MHz, CD_3_OD) spectrum revealed eleven different signals that were assignable. Thus, aromatic signals with AA′BB′ spin patterns were appeared at *δ*_*H*_ 7.19 (2H, *d*, *J* = 8.5 Hz, H-2, 6) and 6.78 (2H, *d*, *J* = 8.5 Hz, H-3, 5) (ring *A*_1_). The signals at *δ*_*H*_ 6.94 (2H, *d*, *J* = 8.5 Hz, H-2′, 6′) and *δ*_*H*_ 6.69 (2H, *d*, *J* = 8.6 Hz, H-3′, 5′) possessing another AA′BB′ spin pattern were assignable to the protons in ring *B*_1_. The signals with a spin pattern AB at *δ*_*H*_ 6.45 (1H, *s,* H-14) and 6.18 (1H, *s,* H-12) correspond to the protons in ring *A*_2_. AB_2_ type protons were also observed at *δ*_*H*_ 6.14 (2H, *s,* H-10′, 14′) and 6.11 (1H, *s,* H-12′) assignable to the aromatic protons in ring *B*_2_. The spectrum also showed an olefinic proton at *δ*_*H*_ 6.25 (1H, *s*, H-7) and sp^3^ methine protons at *δ*_*H*_ 4.19 (1H, *d*, *J* = 2.7 Hz, H-7′) and 3.68 (1H, *d*, *J* = 2.2 Hz, H-8′), revealing the presence of a pentacyclic ring proton (ring C) (Supplementary materials, Table S6).

In its ^13^C NMR spectrum, the compound showed signals at *δ*_*C*_ 159.3 (C-11′, 13′) (ring *B*_2_), 158.4 (C-13) (ring *A*_2_), 157.4 (C-4) (ring *A*_1_), 156.4 (C-4′) (ring *B*_1_), and 155.6 (C-11) (ring A2) attributed to the oxygenated aromatic carbons. The signals at *δ*_*C*_ 150.2 (C-8), 146.2 (C-9′), 143.4 (C-9), 138.1 (C-1′), 130.5 (C-1), and 128.3 (C-10) were assignable to the nonoxygenated quaternary aromatic and olefinic (C-8) carbons of the structure. The spectrum also showed sp^2^ methine aromatic signals at *δ*_*C*_ 130.8 (C-2, 6), 129.1 (C-2′, 6′), 116.1 (C-3, 5), 115.9 (C-3′, 5′), 106.9 (C-10′, 14′), 104.2 (C-12), 103.6 (C-14), and 101.5 (C-12′) and a sp^2^ methine olefinic signal at *δ*_*C*_ 125.6 (C-7). Moreover, two more signals appeared at *δ*_*C*_ 65.1 (C-8′) and 55.6 (C-7′) corresponding to the sp^3^ methine carbons of the pentacyclic ring (ring C). The DEPT-135 spectrum of the compound indicated eight sp^2^ aromatic methine signals, one sp^2^ olefinic methine signal, and two sp^3^ methine signals and was compatible with the ^13^C NMR spectral data of the compound (Supplementary materials, Table S6).

**Figure 3:**
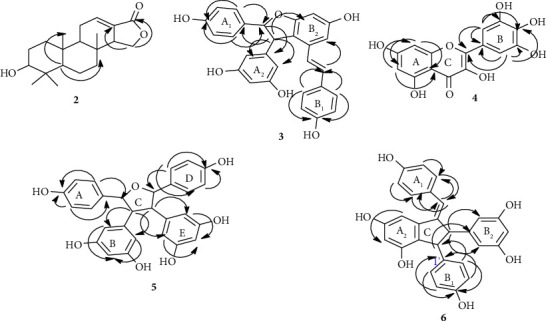
Important HMBC correlations of compounds **2** and **3**–**6**.

The proton linkages and proton-to-carbon connectivity of the compound were verified through bond (COSY) and HMBC correlations, respectively. Thus, the COSY spectrum exhibited ^3^*J* correlations between H-2/6 (*δ*_*H*_ 7.19) and H-3/5 (*δ*_*H*_ 6.78), H-2′/6′ (*δ*_*H*_ 6.94) and H-3′/5′ (*δ*_*H*_ 6.69), and H-7′ (*δ*_*H*_ 4.19) and H-8′ (*δ*_*H*_ 3.67). In addition, the HMBC spectrum showed ^2^*J* and ^3^*J* correlations between H-2/6 (*δ*_*H*_ 7.19) and C-1, C-4, and C-7 (*δ*_*C*_ 130.5, 157.4, and 125.6, respectively), ^3^*J* correlations between H-7 (*δ*_*H*_ 6.25) and C-2/6 (*δ*_*C*_ 130.8), ^3^*J* correlations between H-14 (*δ*_*H*_ 6.45) and C-12 (*δ*_*C*_ 104.2), ^3^*J* correlations between H-2′/6′ (*δ*_*H*_ 6.94) and C-4′, 7′ (*δ*_*C*_ 156.4 and 55.6, respectively), ^3^*J* correlations between H-3′/5′ (*δ*_*H*_ 6.69) and C-1′ (*δ*_*C*_ 138.1), ^2^*J* and ^3^*J* correlations between H-7′ (*δ*_*H*_ 4.19) and C-1′, 9′ (*δ*_*C*_ 138.1 and 146.2, respectively), and ^2^*J* and ^3^*J* correlations between H-8′ (*δ*_*H*_ 3.68) and C-9′, 10′/14′ (*δ*_*C*_ 146.2 and 106.9, respectively) (Figure 3, Supplementary materials, Table S6). Overall, the spectral data were in good coherence with the literature values of parthenocissin A (**6**) [39] (Figure 2).

### 3.2. Antibacterial Activity

Antimicrobial resistance has been recognized among the major threats in folk medicine and food-producing animals [40]. Recent WHO reports also revealed alarming levels of antimicrobial resistance across many parts of the world [41]. To overcome this challenge, we conducted a comprehensive study on four bacterial strains (*E. coli*, *S. aureus*, *P. aeruginosa*, and *S. pyogenes*) to support the traditional relevance of the roots of *C. adenocaule* and introduce alternative sources of medicine. The prevalence of bacterial diseases in Ethiopia and traditional practices of the plant against them motivated us for the selection of the microorganisms.

In the present work, the *in vitro* antibacterial efficacy of the CH_2_Cl_2_ : MeOH (1 : 1) extract and the isolated compounds (**1–6**) of *C. adenocaule* were evaluated against the four bacterial pathogens at four different concentrations (extract: 50, 25, 12.5, and 6.25 mg/mL and isolated compounds: 2, 1, 0.5, and 0.25 mg/mL). For the extract, the highest inhibitory diameters were recorded against *E. coli* (18.00 ± 0.00 mm) and *S. aureus (*17.16 ± 0.24 mm) at 50 mg/mL, compared to ciprofloxacin which showed 21.33 ± 0.47 and 21.50 ± 0.41 mm for *E. coli* and *S. aureus,* respectively, whereas, at the smallest concentration (6.25 mg/mL), the extract also displayed a better inhibition zone against *E. coli* (9.83 ± 0.24 mm) (Table 1). Our findings corroborate earlier research studies wherein the chloroform (22.5 mm) and methanol (15.0 mm) root extracts of *C. adenocaule* exhibited comparable antibacterial activities against *E. coli* and promising zones of diameters were also observed for chloroform (15.0 mm) and methanol (10.5 mm) extracts against *S. aureus*, at unknown concentrations [42]. In the present work, the activity of the extract was also compared to the antibacterial susceptibility of *C. adenocaule* extracts included in the survey of antimicrobial medicinal plants from Uganda [43]. Again, better antibacterial activities were recorded from our findings compared to the report of similar bacterial strains. Based on the data presented, the study established that the CH_2_Cl_2_ : MeOH (1 : 1) root extract of the plant exhibits antibacterial properties against the chosen bacterial strains, which support its traditional relevance.

The activities of the isolated compounds (**1–6**) were also the subjects of this study. At the smallest concentration (0.25 mg*/*mL), the highest inhibitory effect was observed by tricuspidatol A (**5**) (9.83 ± 0.24 mm) against *E. coli* followed by parthenocissin A (**6**) (9.67 ± 0.47 mm) and tricuspidatol A (**5**) (9.33 ± 0.47 mm) against *E. coli* and *S. aureus*, respectively. Furthermore, at 2 mg/mL, the highest inhibitory diameter was also observed for tricuspidatol A (**5**) (16.67 ± 0.47 mm) against *S. aureus* (Table 1).

The antibacterial properties of all the compounds were compared to similar reports of the plant [33]. Accordingly, *β*-sitosterol (**1**), 3-hydroxyisoagatholactone (**2**), *ε-*viniferin (**3**), and tricuspidatol A (**5**) were the subjects of the previous report, and comparable antibacterial activities were reported with the present study. The report revealed the highest inhibitory diameters for *ε-*viniferin (**3**) (12.22 ± 0.74), *β*-sitosterol (**1**) (10.90 ± 1.54), and tricuspidatol A (**5**) (10.79 ± 0.24) against *S. aureus* at 1 mg/mL compared to chloramphenicol standard (11.76 ± 0.77). Regardless of the reference drug, the results were as good as compared with the findings of the present study (Table 1). The previous report also suggested that 3-hydroxy-isoagatholactone (**2**) was identified and tested for the first time and displayed low to moderate antibacterial activities, whereas, in the present work, the activities of the compound were also studied and better results were generated. Many works have been reported on the antimicrobial activities of *β*-sitosterol (**1**) and results revealed that the compound possesses low to moderate activities against different bacterial pathogens [31, 44, 45]. The previous activities were also in good agreement with the findings of the present study. Thus, the *in vitro* antibacterial experimental results support the use of *C. adenocaule* in traditional medicinal systems.

### 3.3. Radical Scavenging Activity

**Figure 4:**
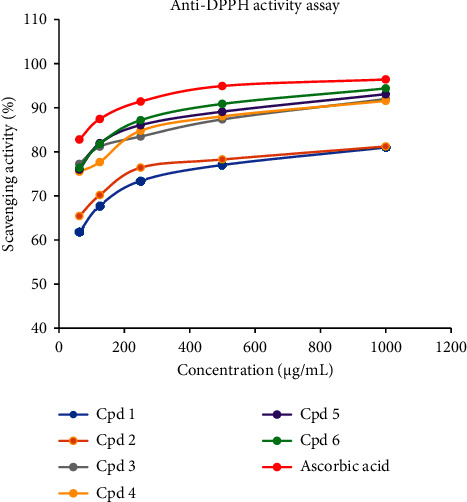
Graphical portrayal of the DPPH radical scavenging power (%) of the compounds of *C. adenocaule* against different concentrations.

The antioxidant properties of all the compounds were evaluated and displayed auspicious results. In the experimental tests, a yellow coloration was observed for most of the prepared concentrations soon after the addition of DPPH solution (0.04 mg/L), indicating the potential radical scavenging capability of the compounds. Results also showed that the activities were dose-dependent and showed smooth correlations with concentration. Accordingly, the strongest activity was displayed by parthenocissin A (**6**) (94.37%) followed by tricuspidatol A (**5**), *ε-*viniferin (**3**), and myricetin (**4**) which revealed 93.07%, 91.97%, and 91.55%, respectively, at 1000 *μ*g/mL, and the results were closer to ascorbic acid (96.40%). At the lowest concentration (62.5 *μ*g/mL), the highest scavenging activity (%) was observed by *ε-*viniferin (**3**) (77.24%) followed by parthenocissin A (**6**) (76.32%), tricuspidatol A (**5**) (76.00%), and myricetin (**4**) (75.49%) compared to ascorbic acid (82.78%). The IC_50_ values of the compounds were also calculated from the relationship curves. Accordingly, *ε-*viniferin (**3**) exhibited the strongest IC_50_ value (0.32 *μ*g/mL) followed by tricuspidatol A (**5**), parthenocissin A (**6**), and myricetin (**4**) (0.67, 0.98, and 1.05 *μ*g/mL, respectively). The calculation results were not very far from the IC_50_ value of ascorbic acid (0.075 *μ*g/mL). In contrast, the weakest IC_50_ value was recorded by *β*-sitosterol (**1**) (9.87 *μ*g/mL) which was far apart from the standard (Table 2 and Figure 4). Generally, the radical scavenging activity of the compounds can be related to their proton-donating capabilities. As a result, *ε-*viniferin (**3**), myricetin (**4**), tricuspidatol A (**5**), and parthenocissin A (**6**), which are comprised of many proton-donating sites, showed strong scavenging activities.

The IC_50_ values were calculated from the logarithmic regressions of the relationship curves on a Microsoft Excel 2016 spreadsheet. Accordingly, the trend line analyses of the compounds generated the following equations, and the values were calculated using logarithmic relationships (Figure 4). *β*-Sitosterol (**1**): *Y* = 6.8687ln(*x*) + 34.269, *R*^2^ = 0.9873; 3-hydroxy isoagatholactone (**2**): *Y* = 5.7188ln(*x*) + 42.722, *R*^2^ = 0.9809; *ε-*viniferin (**3**): *Y* = 5.1418ln(*R*^2^) + 55.85, *R*^2^ = 0.9989; myricetin (**4**): *Y* = 6.1315ln(*x*) + 49.647, *R*^2^ = 0.9848; tricuspidatol A (**5**): *Y* = 5.9569ln(*x*) + 52.345, *R*^2^ = 0.9850; parthenocissin A (**6**): *Y* = 6.5195ln(*x*) + 50.101, *R*^2^ = 0.9885; and ascorbic acid: *Y* = 5.009ln(*x*) + 62.933, *R*^2^ = 0.9914, and the *Y* values were labeled as 50.

### 3.4. Molecular Docking Studies

In the present study, the protein targets were selected based on their metabolic importance to the microorganisms, the respective strains' *in vitro* antibacterial and antioxidant activities, and the similarity of the compound to the cocrystalized ligands in the protein complex. In the PDB database, proteins are found in complexes with ligands (cocrystallization). Target proteins are prepared from the cocrystallized structures by removing water molecules, identifying the appropriate binding sites, and adding polar hydrogens. The prepared proteins must have a suitable binding pocket/active site into which a drug or drug-like molecule can bind, and the structures of the cocrystallized ligands play prominent factors in determining similarities with the isolated secondary metabolite candidates for docking. Most of the protein complexes in the PDB database are made up of multiple ligands and active sites. Thus, a cocrystallized ligand having related structural features with our candidate compounds suggests that the active site of that specific protein is a promising candidate to be considered for molecular docking study, and hence, the respective proteins are given prior consideration.

**Figure 5:**
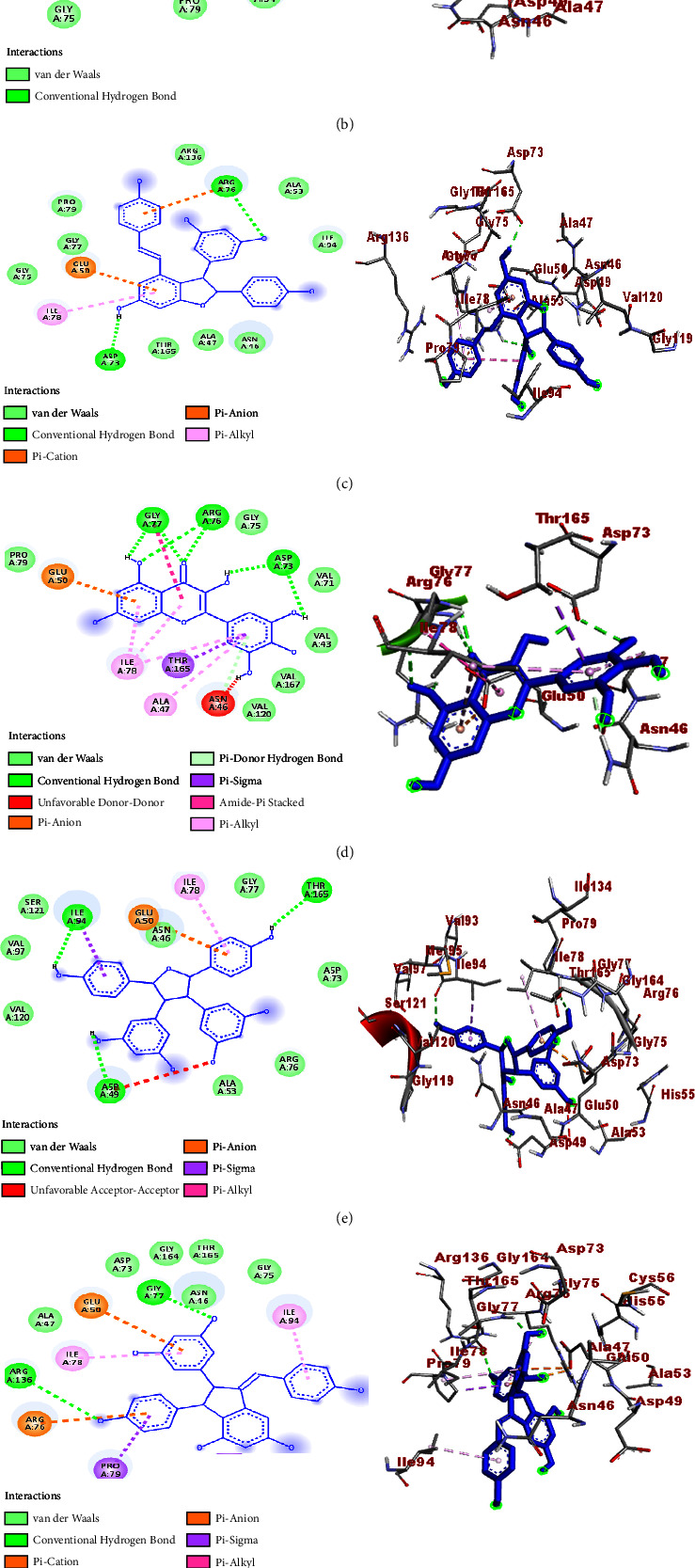
2D (left) and 3D (right) binding interactions of compounds **1–6** and ciprofloxacin against DNA gyrase B (*E. coli*). (a) Compound **1**. (b) Compound **2**. (c) Compound **3**. (d) Compound **4**. (e) Compound **5**. (f) Compound **6**. (g) Ciprofloxacin.

**Figure 6:**
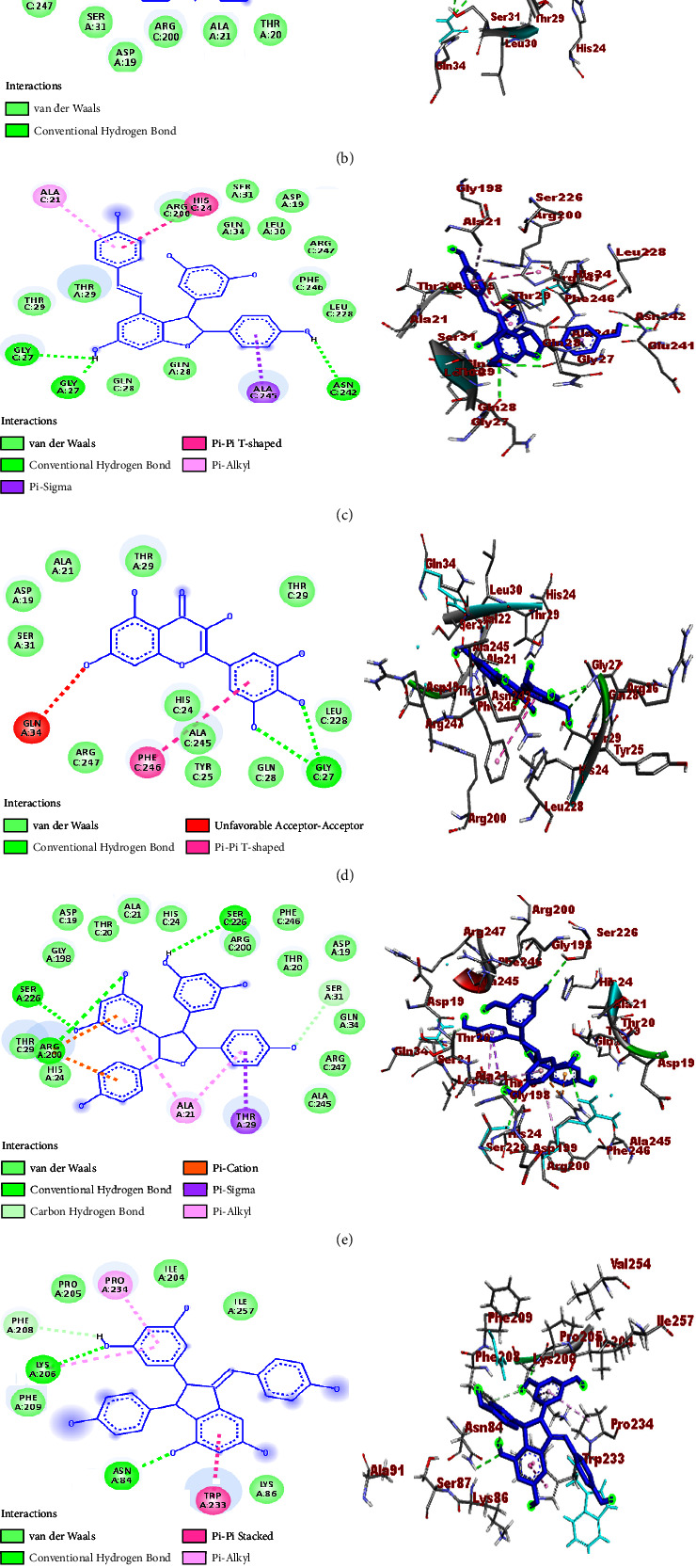
2D (left) and 3D (right) binding interactions of compounds **1–6** and ciprofloxacin against the N-terminal domain of PqsA. (a) Compound **1**. (b) Compound **2**. (c) Compound **3**. (d) Compound **4**. (e) Compound **5**. (f) Compound **6**. (g) Ciprofloxacin.

**Figure 7:**
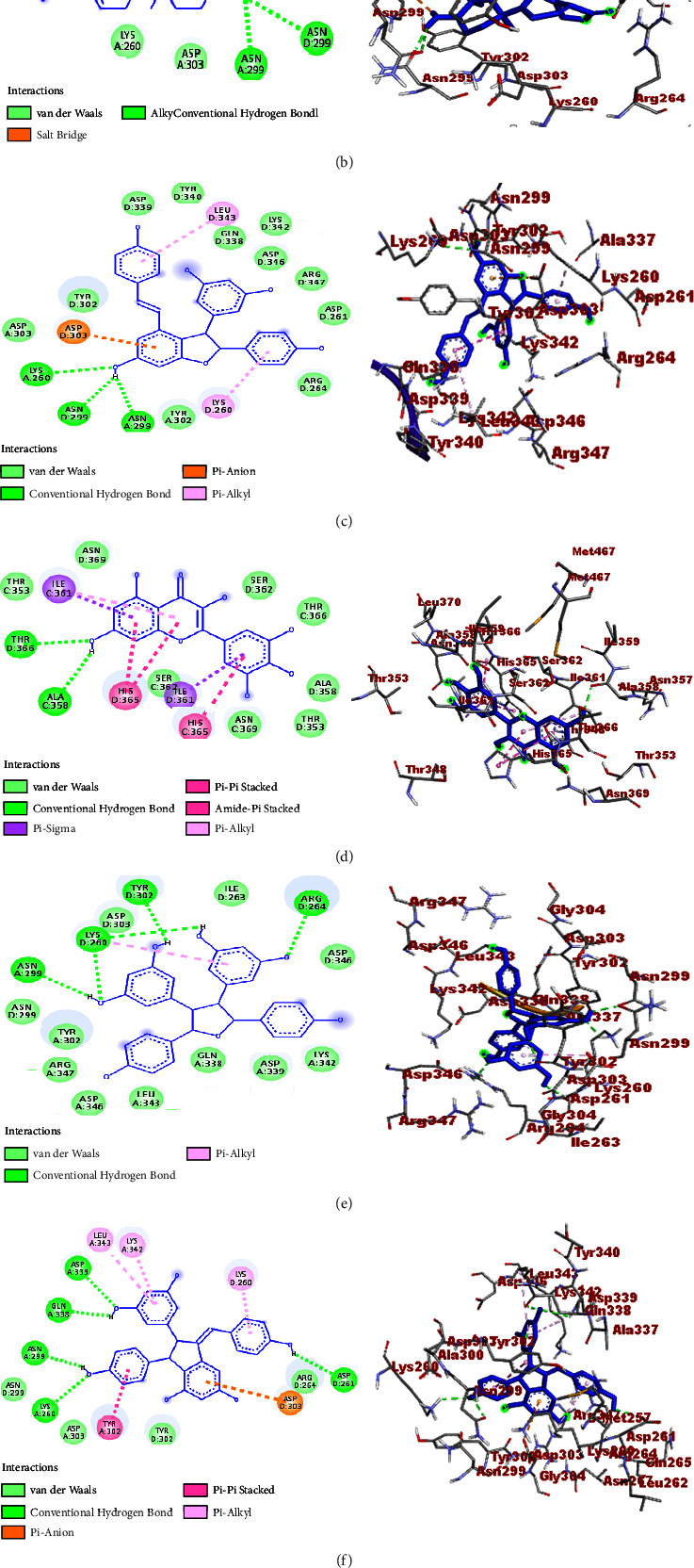
2D (left) and 3D (right) binding interactions of compounds **1–6** and ciprofloxacin against PK (*S. aureus*). (a) Compound **1**. (b) Compound **2**. (c) Compound **3**. (d) Compound **4**. (e) Compound **5**. (f) Compound **6**. (g) Ciprofloxacin.

**Figure 8:**
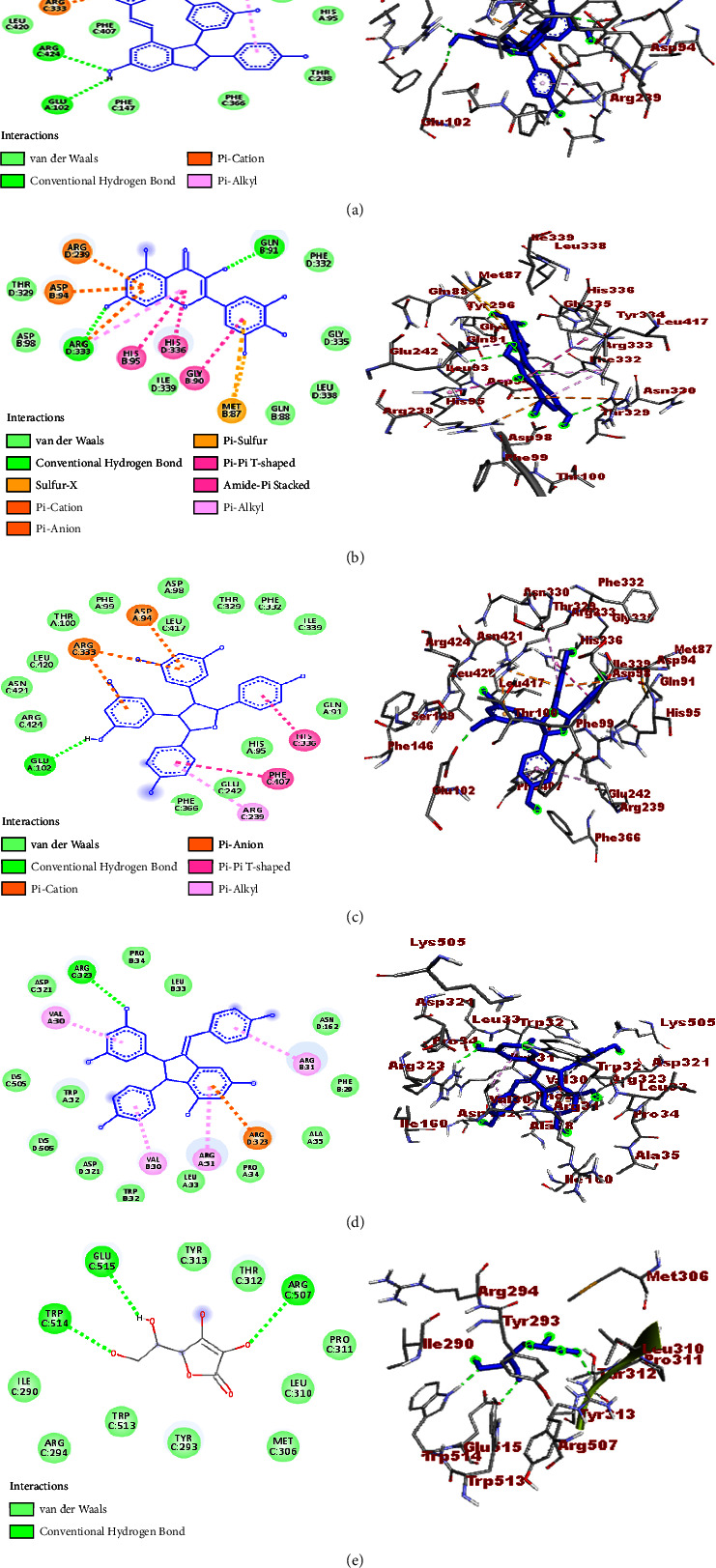
2D (left) and 3D (right) binding interactions of compounds **3–6** and ascorbic acid against human myeloperoxidase. (a) Compound **3**. (b) Compound **4**. (c) Compound **5**. (d) Compound **6**. (e) Ascorbic acid.

**Figure 9:**
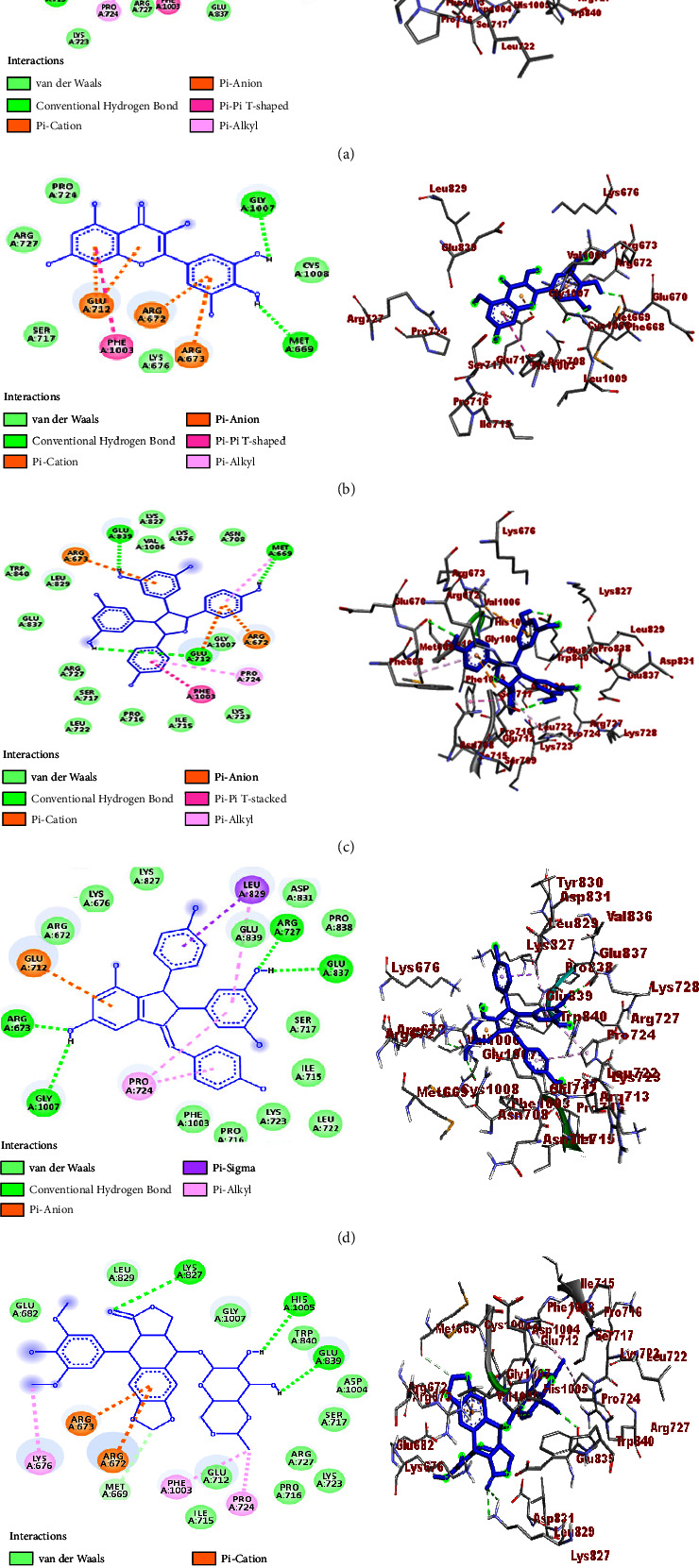
2D (left) and 3D (right) binding interactions of compounds **3–6** and etoposide against topoisomerase II *α*. (a) Compound **3**. (b) Compound **4**. (c) Compound **5**. (d) Compound **6**. (e) Etoposide.

A molecular docking study was performed to establish the binding affinity and binding interactions of the compounds (**1–6**) against protein targets of three bacterial pathogens. DNA gyrase B of *E. coli* (PDB ID: 6F86), pyruvate kinase (PK) of *S. aureus* (PDB ID: 3T07), and PqsA of *P. aeruginosa* (PDB ID: 5OE3) were used to consolidate the *in vitro* experimental results. The molecular docking properties of the compounds (**3–6**) were also studied against human myeloperoxidase (PDB ID: 1DNU) and topoisomerase II *α* (PDB ID: 4FM9), and their respective binding affinity, H-bond, and residual interactions were reported in both tabular and figure forms (Tables 3–7 and Figures 5–9). The docking results exhibited binding affinities ranging from −7.4 to −6.6, −9.2 to 7.1, −8.7 to −7.9, −7.9 to −6.9, and −9.9 to −7.5 kcal/mol against DNA gyrase B, PqsA, PK, human myeloperoxidase, and topoisomerase II *α* protein targets, respectively. The most stable binding energy was observed for *ε-*viniferin (**3**) (−9.9 kcal/mol) against topoisomerase II *α*, followed by *ε-*viniferin (**3**) and parthenocissin A (**6**) (−9.2 kcal/mol each) against PqsA and topoisomerase II *α*, respectively.

#### 3.4.1. Molecular Docking Study against DNA Gyrase B (*E. coli*)

The molecular docking process against DNA gyrase B (*E. coli*) revealed binding affinities of −7.4, −7.3, −7.1,−6.9, and −6.6 kcal/mol for tricuspidatol A (**5**), parthenocissin A (**6**), (*ε*-viniferin (**3**) and myricetin (**4**)), 3-hydroxy isoagatholactone (**2**), and *β-*sitosterol (**1**), respectively, and all the compounds displayed smaller binding affinity than ciprofloxacin (−7.6 kcal/mol). This was consistent with the *in vitro* experimental results of the compounds (Table 1). Small values of binding affinities are always related to good stability and hence improved binding interaction of compounds/ligands with protein binding sites. Accordingly, tricuspidatol A (**5**) displayed greater binding affinity (−7.4 kcal/mol) against DNA gyrase B than the others (Table 3). The H-bond and residual amino acid interactions of the compounds along with ciprofloxacin were also clearly stated. H-bond interactions were observed between 3-hydroxyisoagatholactone (**2**) and Gly-77, *ε*-viniferin (**3**) and Asp-73 and Arg-76, myricetin (**4**) and Arg-76, Gly-77 and Asp-73, tricuspidatol A (**5**) and Thr-165, Ile-94 and Asp-49, and parthenocissin A (**6**) and Gly-77 and Arg-136, of which *ε*-viniferin (**3**) and myricetin (**4**) shared similar H-bond interactions with ciprofloxacin (Arg-76, Asn-46, and Asp-73) (Table 3 and Figure 5).

#### 3.4.2. Molecular Docking Study against *P. aeruginosa* PqsA

The binding interactions of the isolated compounds against *P. aeruginosa* PqsA were also consistent with their *in vitro* antibacterial activities (Table 1). The highest binding affinity was displayed by *ε*-viniferin (**3**) (−9.2 kcal/mol), followed by parthenocissin A (**6**) and myricetin (**4**) (−9.0 and −8.8 kcal/mol, respectively). All the compounds except *β-*sitosterol (**1**) showed H-bond interactions with at least one amino acid residue. Thus, 3-hydroxyisoagatholactone (**2**) showed H-bond interaction with Gln-34, *ε*-viniferin (**3**) with Asn-242 and Gly-27, myricetin (**4**) with Gly-27, tricuspidatol A (**5**) with Arg-200, Ser-226, and Ser-31, and parthenocissin A (**6**) with Asn-84, Lys-206, and Phe-208, and more than two H-bond interactions were displayed by tricuspidatol A (**5**) and parthenocissin A (**6**) with better binding interaction than ciprofloxacin which formed two H-bond interactions with Arg-200 and Asp-19 (Table 4 and Figure 6). *β-*Sitosterol (**1**) failed to show H-bond interactions against *P. aeruginosa* PqsA residual amino acids. This suggested that the binding affinities of the compound account for other amino acid interactions where no H-bonds are observed.

#### 3.4.3. Molecular Docking Study against *S. aureus* PK

In the present work, the ligand-*S. aureus* PK interactions of the isolated compounds were also established and tricuspidatol A (**5**) exhibited maximum binding affinity (−8.7 kcal/mol) followed by parthenocissin A (**6**) (−8.5 kcal/mol) and myricetin (**4**) (−8.3 kcal/mol). The binding affinities of the compounds were almost closer to the ciprofloxacin standard (−8.9 kcal/mol) and agreed with the experimental *in vitro* activities (Table 1). The molecular docking scores also showed H-bond interactions between *β-*sitosterol (**1**) and Ala-337, 3-hydroxyisoagatholactone (**2**) and Asp-303 and Asn-29, *ε*-viniferin (**3**) and Lys-260 and Asn-299, myricetin (**4**) and Thr-366 and Ala-358, tricuspidatol A (**5**) and Lys-260, Arg-264, Asn-299, and Tyr-302, and parthenocissin A (**6**) and Lys-260, Asp-339, Gln-338, Asn-299, and Asp-261 (Table 5 and Figure 7).

#### 3.4.4. Molecular Docking Study against Human Myeloperoxidase

The *in vitro* antioxidant capabilities of four compounds (**3–6**) were confirmed with a molecular docking study against the human myeloperoxidase (PDB: 1DNU) target. The binding affinities were recorded in the range of −7.9 kcal/mol (*ε-*viniferin (**3**)) to −6.9 kcal/mol (myricetin (**4**)), and the docking scores were less than ascorbic acid (−8.1 kcal/mol). This was consistent with the *in vitro* experimental results of the compounds (Table 2). The H-bond and residual amino acid report showed that all the investigated compounds exhibited H-bond interactions with at least one amino acid. Accordingly, H-bond interactions were observed by *ε-*viniferin (**3**) with Arg-424, Asp-94, and Glu-102, myricetin (**4**) with Gln-91 and Arg-333, tricuspidatol A (**5**) with Glu-102, and parthenocissin A (**6**) with Arg-323 (Table 6 and Figure 8).

#### 3.4.5. Molecular Docking Study against Topoisomerase II *α*

Notably, the uses of *C. adenocaule* against various types of cancer cells have been reported. Matata and co-workers reported that the root extracts of the plant exhibit cytotoxic properties against HeLa cervical cancer cell lines [46]. According to the report, the ethyl acetate fractions showed high toxicity activities against the HeLa cervical cancer cells with IC_50_ = 3.4 ± 0.3 *μ*g/mL. Similarly, the methanol and ethyl acetate fractions exhibited moderate and high toxicity, respectively, against *Artemia nauplii* with higher toxicity than cyclophosphamide standard supporting cytotoxic properties against cancer cell lines [46]. Thus, inspired by its considerable anticancer properties, the *in silico* molecular docking profile of four compound isolates (**3–6**) was computed against the topoisomerase II *α* (PDB ID: 4FM9) protein target, and promising results were recorded. *ε*-Viniferin (**3**) showed the maximum binding affinity (−9.9 kcal/mol) followed by parthenocissin A (**6**) and tricuspidatol A (**5**) (−9.2 and −8.7 kcal/mol, respectively). *ε*-Viniferin (**3**) also depicted greater binding affinity than etoposide (−9.4 kcal/mol), hence good binding interactions. The ligand-topoisomerase II *α* binding interactions were also stabilized through the formation of H-bonds between *ε*-viniferin (**3**) and Asp-831, Glu-839, His-1005, Glu-712, and Ile-715, myricetin (**4**) and Gly-1007 and Met-669, tricuspidatol A (**5**) and Met-669, Glu-839, and Glu-712, and parthenocissin A (**6**) and Arg-673, Gly-1007, Arg-727, and Glu-837 accounting proportional number of H-bonds with etoposide (Lys-827, Glu-839, His-1005, and Met-669 (C-H bond)) (Table 7 and Figure 9).

### 3.5. *In Silico *Drug-Likeness Predictions

The *in silico* drug-likeness properties of all the compounds were established based on Lipinski's rule of five [27]. The canonical SMILEs of each compound were generated from the respective chemical structures and uploaded to the SwissADME tool to produce the pharmacokinetic and physicochemical profiles of the compounds. The estimation method indicated that 3-hydroxyisoagatholactone (**2**) and *ε*-viniferin (**3**) fulfill Lipinski's rule with zero violation and were in good agreement with the ciprofloxacin standard (no violation) (Table 8). The prediction outcomes also revealed that all the compounds generated lipophilicity (iLogP) values ranging from 1.08 to 4.79 (<5) showing their optimum lipophilicity. The NRB values of all the compounds (<7) also showed their conformational stability [47]. The total TPSA values which are used to predict the bioavailability and permeability of the compounds were in the range of 20.23–151.59 Å^2^. Thus, except myricetin (**4**), all the compounds displayed lower TPSA values than the cutoff value (140 Å^2^), revealing their safe absorption in the intestine.

### 3.6. ADMET Properties

In the present work, we investigated the *in silico* pharmacological screening predictions (ADME) of the compounds with the help of the SwissADME tool. The important ADME properties related to the pharmacokinetic profile of the compounds are displayed in Table 9. The log Kp values of the compounds were in the range of −7.40 (myricetin (**4**)) up to −2.20 cm/s (*β-*sitosterol (**1**)). Literature surveys revealed that the smaller the logKp, the lesser the skin permeability of the molecule [28]. Thus, myricetin (**4**) and tricuspidatol A (**5**) displayed promising logKp values (−7.40 and −6.14 cm/s, respectively) closer to ciprofloxacin (−9.09 cm/s). High values of the blood-brain barrier (BBB) and gastrointestinal absorption are also related to the safe absorption and distribution of drug molecules [47]. Accordingly, 3-hydroxyisoagatholactone (**2**) and *ε*-viniferin (**3**) displayed high GIA values and hence good absorption in the intestine. The prediction tool also exhibited that all the investigated compounds generated negative (No) P-gp substrate values and hence noninhibitors of most of the evaluated cytochrome enzymes. Inhibition interactions were verified by myricetin (**4**) against CYP1A2, 3-hydroxyisoagatholactone (**2**), and *ε*-viniferin (**3**) against CYP2C9 and myricetin (**4**), and tricuspidatol A (**5**) against CYP3A4.

The toxicological classifications of drugs/chemicals are convoyed in terms of LD_50_ (median lethal dose) which is the amount of drug that is lethal to one-half (50%) of the experimental organisms viable to it [48]. Acute toxicity studies are performed to have safer routes of exposure (i.e., inhalation, dermal, and oral) and rodents are mostly employed to evaluate the lethal dose [49]. According to EPA's 4 categories of hazard classifications, compounds/chemicals with LD_50_ ≤ 50 mg/kg are highly toxic, 50 < LD_50_ ≤ 500 mg/kg (moderately toxic), 500 < LD_50_ ≤ 5000 mg/kg (slightly toxic) and LD_50_ > 5000 mg/kg (safe chemicals) [49]. Hence, all the investigated compounds showed no acute toxicity (Table 10). The prediction outcomes also revealed that all the compounds showed no cytotoxicity and hepatotoxicity properties. This was consistent with the toxicity prediction values of ciprofloxacin. *ε*-Viniferin (**3**) and myricetin (**4**) were active towards carcinogenicity, *β-*sitosterol (**1**), 3-hydroxy isoagatholactone (**2**), *ε*-viniferin (**3**), and parthenocissin A (**6**) showed immunotoxicity, and only myricetin (**4**) was active towards mutagenicity. Thus, based on the prediction methods, the compounds of study possess drug candidate properties.

## 4. Conclusion

The emergence of novel pathogens and the growth of antibiotic resistance raise momentous apprehensions in the healthcare industry. In this study, six compounds were isolated from *C. adenocaule* (roots) and their *in vitro* and *in silico* antioxidant and antibacterial properties were studied. The antibacterial tests showed that the compounds exhibit valuable activities, and at the smallest concentration (0.25 mg/mL), the highest inhibition zones were displayed by tricuspidatol A (**5**) (9.83 ± 0.24) and parthenocissin A (**6**) (9.67 ± 0.47) against *E. coli,* which were smaller than the reference drug (ciprofloxacin) but unquestionably significant. The antioxidant efficacy of the compounds was also promising. *ε-*Viniferin (**3**) (IC_50_ = 0.32 *μ*g/mL) exhibited the strongest DPPH radical scavenging activity compared to the others. The binding affinities of the compounds were also consistent with the *in vitro* experimental studies. *ε*-Viniferin (**3**) (−9.9 kcal/mol) displayed the highest binding affinity against topoisomerase II *α* protein target. The toxicity predictions showed no cytotoxicity and hepatotoxicity effects for all the compounds consolidating their drug likeness properties. Thus, the CH_2_Cl_2_ : MeOH (1 : 1) extract of *C. adenocaule* is suggested as a valuable source of natural antibacterial and antioxidant compounds, contributing opportunities for emerging novel pharmaceuticals, which also supports the traditional uses of *C. adenocaule*. We also suggest further efforts to attempt the plant's phytochemistry to isolate novel compounds with additional biological activities that are not included in this study.

## Figures and Tables

**Table 1:** Inhibition zone (mean ± SD, in mm) of CH_2_Cl_2_ : MeOH (1 : 1) extract and the isolated compounds (**1**–**6**).

Bacterial strain	Conc. (mg/mL)	Average inhibition zone (mm) (3 trials)							
		CH_2_Cl_2_ : MeOH extract	**1**	**2**	**3**	**4**	**5**	**6**	Ciprofloxacin (30 *μ*g/disc)
*E. coli*	2	18.00 ± 0.00	12.67 ± 0.47	13.50 ± 0.87	14.30 ± 0.47	14.67 ± 0.47	16.17 ± 0.24	15.33 ± 0.24	21.33 ± 0.47
	1	14.50 ± 0.41	10.50 ± 0.41	11.33 ± 0.47	11.67 ± 0.47	12.00 ± 0.00	13.50 ± 0.41	12.50 ± 0.41	
	0.5	11.50 ± 0.41	9.33 ± 0.47	9.50 ± 0.00	9.83 ± 0.24	10.00 ± 0.00	11.33 ± 0.47	10.50 ± 0.41	
	0.25	9.83 ± 0.24	7.00 ± 0.00	7.67 ± 0.47	8.83 ± 0.24	9.00 ± 0.00	9.83 ± 0.24	9.67 ± 0.47	

*S. aureus*	2	17.16 ± 0.24	11.83 ± 0.24	13.33 ± 0.47	14.00 ± 0.00	14.33 ± 0.47	16.67 ± 0.47	15.33 ± 0.47	21.50 ± 0.41
	1	13.83 ± 0.24	9.83 ± 0.24	10.16 ± 0.24	11.33 ± 0.47	11.50 ± 0.41	12.67 ± 0.47	11.67 ± 0.47	
	0.5	11.00 ± 0.00	8.16 ± 0.24	8.50 ± 0.41	9.33 ± 0.47	9.50 ± 0.41	10.00 ± 0.00	9.67 ± 0.47	
	0.25	9.50 ± 0.41	NA	7.33 ± 0.47	8.16 ± 0.24	8.33 ± 0.47	9.33 ± 0.47	8.83 ± 0.24	

*P. aeruginosa*	2	15.67 ± 0.47	12.00 ± 0.00	12.50 ± 0.41	14.16 ± 0.24	13.16 ± 0.24	12.67 ± 0.47	13.83 ± 0.24	20.00 ± 0.81
	1	12.00 ± 0.00	9.33 ± 0.47	10.16 ± 0.24	11.50 ± 0.41	10.83 ± 0.24	10.33 ± 0.47	11.16 ± 0.24	
	0.5	10.16 ± 0.24	8.00 ± 0.00	8.83 ± 0.24	10.00 ± 0.00	9.33 ± 0.47	9.16 ± 0.24	9.50 ± 0.41	
	0.25	8.50 ± 0.00	NA	7.16 ± 0.24	8.33 ± 0.47	7.67 ± 0.47	7.33 ± 0.47	8.00 ± 0.00	

*S. pyogenes*	2	16.33 ± 0.47	12.50 ± 0.41	12.83 ± 0.24	15.33 ± 0.47	14.50 ± 0.00	13.67 ± 0.47	13.83 ± 0.24	21.00 ± 0.81
	1	13.16 ± 0.24	9.67 ± 0.47	10.00 ± 0.00	11.67 ± 0.47	11.33 ± 0.47	10.67 ± 0.47	10.83 ± 0.24	
	0.5	10.83 ± 0.24	8.33 ± 0.47	8.67 ± 0.47	10.16 ± 0.24	9.50 ± 0.00	9.00 ± 0.00	9.16 ± 0.24	
	0.25	9.50 ± 0.41	7.16 ± 0.24	7.33 ± 0.47	9.00 ± 0.81	8.67 ± 0.47	7.83 ± 0.24	8.00 ± 0.81	

CH_2_Cl_2_, dichloromethane; MeOH, methanol; NA, no activity; SD, standard deviation. For CH_2_Cl_2_ : MeOH (1 : 1) extract, 2: 50 mg/mL, 1: 25 mg/mL, 0.5: 12.5 mg/mL, and 0.25: 6.25 mg/mL.

**Table 2:** DPPH radical scavenging assay (%) of the isolated compounds (**1–6**).

Conc. (*μ*g/mL)	**1**	**2**	**3**	**4**	**5**	**6**	Ascorbic acid
62.5	61.79	65.43	77.24	75.49	76.00	76.32	82.78
125	67.78	70.18	81.17	77.66	81.95	81.77	87.44
250	73.41	76.41	83.47	84.77	86.06	87.17	91.41
500	77.01	78.26	87.35	88.04	89.10	90.86	94.92
1000	80.98	81.21	91.97	91.55	93.07	94.37	96.40
IC_50_ (*μ*g/mL)	9.87	3.56	0.32	1.05	0.67	0.98	0.075

**Table 3:** Molecular docking study of the isolated compounds (**1–6**) and ciprofloxacin against DNA gyrase B (*E. coli*).

Compound/ligand	Binding affinity (kcal/mol)	H-bond	Residual amino acid interactions	
			Hydrophobic/electrostatic/halogen	Van der Waals
**1**	−6.6	—	Pro-79, Ile-78, Val-71, Ala-47, Val-43, Val-167, and Val-120	Asn-46, Asp-73, Ile-94, Glu-50, Thr-165, and Arg-76
**2**	−6.9	Gly-77	—	Asn-46, Glu-50, Asp-49, Asp-73, Thr-165, Gly-75, Ile-78, Pro-79, and Ile-94
**3**	−7.1	Asp-73 and Arg-76	Arg-76, Glu-50, and Ile-78	Ile-94, Ala-53, Arg-136, Pro-79, Gly-77, Gly-75, Thr-165, Ala-47, and Asn-46
**4**	−7.1	Arg-76, Gly-77, and Asp-73	Glu-50, Thr-165, Gly-77, Ile-78, and Ala-47	Pro-79, Gly-75, Val-71, Val-43, Val-167, and Val-120
**5**	−7.4	Thr-165, Ile-94, and Asp-49	Glu-50, Ile-78, and Ile-94	Val-120, Val-97, Ser-121, Asn-46, Gly-77, Asp-73, Arg-76, and Ala-53
**6**	−7.3	Gly-77 and Arg-136	Arg-76, Glu-50, Pro-79, Ile-78, and Ile-94	Ala-47, Asp-73, Gly-164, Thr-165, Asn-46, and Gly-75
Ciprofloxacin	−7.6	Asp-73, Asn-46, and Arg-76	Glu-50, Arg-76, Gly-77 Asn-46, Ile-94, and Ile-78	Pro-79, Thr-165, and Ala-47

**Table 4:** Molecular docking study of the isolated compounds (**1–6**) and ciprofloxacin against the N-terminal domain of PqsA.

Compound/ligand	Binding affinity (kcal/mol)	H-bond	Residual amino acid interactions	
			Hydrophobic/electrostatic	Van der Waals
**1**	−7.1	—	Trp-233, Pro-234, and Val-254	Ile-204, Ile-257, Ala-256, Pro-205, Lys-206, Phe-208, Pro-255, Ser-280, Gly-279, and Phe-209
**2**	−8.5	Gln-34	—	Ala-245, Phe-246, Arg-247, Ser-31, Asp-19, Arg-200, Ala-21, Thr-20, Thr-29, and His-24
**3**	−9.2	Gly-27 and Asn-242	Ala-21, Ala-245, and His-24	Leu-228, Thr-29, Gln-28, Phe-246, Arg-247, Gln-34, Leu-30, Asp-19, Ser-31, and Arg-200
**4**	−8.8	Gly-27	Phe-246	Ser-31, Asp-19, Arg-247, His-24, Ala-245, Tyr-25, Gln-28, Leu-228, Thr-29, and Ala-21
**5**	−8.7	Arg-200, Ser-226, and Ser-31	Ala-21, Arg-200, and Thr-29	Ala-245, Arg-247, Gln-34, Asp-19, Thr-20, Phe-246, Arg-200, His-24, Ala-21, Gly-198, and Thr-29
**6**	−9.0	Phe-208, Asn-84, and Lys-206	Trp-233, Lys-206, Pro-234	Lys-86, Ile-257, Phe-209, Pro-205, and Ile-204
Ciprofloxacin	−9.3	Asp-19 and Arg-200	His-24 and Ala-21	Asp-19, Ser-31, Ala-21, Ser-31, Thr-20, Thr-29, and Arg-200

**Table 5:** Molecular docking study of the isolated compounds (**1–6**) and ciprofloxacin against PK (*S. aureus*).

Compound/ligand	Binding affinity (kcal/mol)	H-bond	Residual amino acid interactions	
			Hydrophobic/electrostatic	Van der Waals
**1**	−7.9	Ala-337	Lys-260 and Tyr-302	Asp-303, Lys-260, Gln-338, Asp-339, and Lys-342
**2**	−8.0	Asp-303, salt bridge; attractive charge, Asn-29	—	Arg-264, Lys-342, Tyr-302, Lys-260, and Asp-303
**3**	−8.2	Lys-260 and Asn-299	Asp-303, Lys-260, and Leu-343	Asp-339, Tyr-340, Lys-342, Gln-338, Asp-346, Arg-347, Asp-261, Arg-264, Tyr-302, Asp-303, and Tyr-302
**4**	−8.3	Thr-366 and Ala-358	Ile-361 and His-365	Thr-353, Asn-369, Ser-362, Thr-366, and Ala-358
**5**	−8.7	Lys-260, Arg-264, Asn-299, and Tyr-302	Lys-260	Asp-346, Ile-263, Asp-303, Asn-299, Tyr-302, Arg-347, Leu-343, Gln-338, Asp-339, and Lys-342
**6**	−8.5	Lys-260, Asp-339, Gln-338, Asn-299, and Asp-261	Asp-303, Tyr-302, Lys-342, Leu-343, and Lys-260	Asn-299, Asp-303, Tyr-302, and Arg-264
Ciprofloxacin	−8.9	Ser-362, Thr-366, and Asn-369	His-365 and Ile-361	Ala-358, Asn-369, Thr-348, Ile-361, and Met-467

**Table 6:** Molecular docking study of the isolated compounds (**3–6**) and ascorbic acid against human myeloperoxidase.

Compound/ligand	Binding affinity (kcal/mol)	H-bond	Residual amino acid interactions	
			Hydrophobic/electrostatic	Van der Waals
**3**	−7.9	Arg-424, Asp-94, and Glu-102	Arg-239 and Arg-333	Phe-147, Phe-366, Thr-238, His-95, Glu-242, Gln-91, Phe-99, Leu-406, Leu-417, Phe-332, Thr-329, Asp-98, Thr-100, Leu-420, and Phe-407
**4**	−6.9	Gln-91 and Arg-333	Met-87, Arg-239, Arg-333, Asp-94, His-95, His-336, and Gly-90	Gln-88, Leu-338, Gly-335, Phe-332, Thr-329, Asp-98, and Ile-339
**5**	−7.7	Glu-102	Arg-333, Asp-94, His-336, Phe-407, and Arg-239	Phe-366, Glu-242, His-95, Gln-91, Ile-339, Phe-332, Thr-329, Asp-98, Leu-417, Phe-99, Thr-100, Leu-420, Asn-421, and Arg-424
**6**	−7.5	Arg-323	Arg-323, Arg-31, and Val-30	Asp-321, Pro-34, Leu-33, Asn-162, Phe-29, Ala-35, Trp-32, and Lys-505
Ascorbic acid	−8.1	Trp-514, Glu-515, and Arg-507	—	Ile-290, Arg-294, Trp-513, Tyr-293, Met-306, Leu-310, Pro-311, Thr-312, and Tyr-313

**Table 7:** Molecular docking study of the isolated compounds (**3–6**) and etoposide against topoisomerase II *α*.

Compound/ligand	Binding affinity (kcal/mol)	H-bond	Residual amino acid interactions	
			Hydrophobic/electrostatic	Van der Waals
**3**	−9.9	Asp-831, Glu-839, His-1005, Glu-712, and Ile-715	Arg-672, Glu-712, Glu-839, Phe-1003 Pro-724, and Leu-829	Met-669, Arg-673, Gly-1007, Asn-708, Lys-676, Trp-840, Lys-728, Glu-837, Arg-727, Lys-723, Pro-716, and Ser-717
**4**	−7.5	Gly-1007 and Met-669	Arg-672, Arg-673, Glu-712, and Phe-1003	Pro-724, Arg-727, Ser-717, Lys-676, and Cys-1008
**5**	−8.7	Met-669, Glu-839, and Glu-712	Arg-672, Arg-673, Glu-712, Phe-1003, Met-669, and Pro-724	Trp-840, Leu-829, Glu-837, Arg-727, Ser-717, Leu-722, Pro-716, Lys-723, Ile-715, Gly-1007, Asn-708, Lys-676, Val-1006, and Lys-827
**6**	−9.2	Arg-673, Gly-1007, Arg-727, and Glu-837	Leu-829, Pro-724, and Glu-712	Pro-838, Glu-839, Asp-831, Lys-827, Lys-676, Arg-672, Phe-1003, Lys-723, Pro-716, Leu-722, Ile-715, and Ser-717
Etoposide	−9.4	Lys-827, Glu-839, His-1005, and Met-669 (C-H bond)	Arg-672, Arg-673, Pro-724, Lys-676, and Phe-1003	Glu-682, Leu-829, Gly-1007, Trp-840, Asp-1004, Ser-717, Arg-727, Lys-723, Pro-716, Glu-712, and Ile-715

**Table 8:** *In silico* drug-likeness properties of the isolated compounds (**1–6**) generated by SwissADME.

Compound no.	MF	MW (g/mol)	NRB	NHA	NHD	LogP (iLogP)	TPSA (Å^2^)	Violation (Lipinski's rule of five)
**1**	C_29_H_50_O	414.71	6	1	1	4.79	20.23	1
**2**	C_20_H_30_O_3_	318.45	0	3	1	2.92	46.53	0
**3**	C_28_H_22_O_6_	454.47	4	6	5	2.41	110.38	0
**4**	C_15_H_10_O_8_	318.24	1	8	6	1.08	151.59	1
**5**	C_28_H_24_O_7_	472.49	4	7	6	2.04	130.61	1
**6**	C_28_H_22_O_6_	454.47	3	6	6	2.25	121.38	1
Ciprofloxacin	C_17_H_18_FN_3_O_3_	331.34	3	5	2	2.24	74.57	0

MF, molecular formula; MW, molecular weight; NHA, number of hydrogen acceptors; NHD, number of hydrogen donors; NRB, number of rotatable bonds; TPSA, total polar surface area.

**Table 9:** ADME predictions of the isolated compounds (**1–6**) generated by SwissADME computing tool.

Compound no.	LogKp (cm/s)	GIA	BBB	Inhibitor interaction					
				P-gp substrate	CYP1A2	CYP2C19	CYP2C9	CYP2D6	CYP3A4
**1**	−2.20	Low	No	No	No	No	No	No	No
**2**	−5.23	High	Yes	No	No	No	Yes	No	No
**3**	−5.24	High	No	No	No	No	Yes	No	No
**4**	−7.40	Low	No	No	Yes	No	No	No	Yes
**5**	−6.14	Low	No	No	No	No	No	No	Yes
**6**	−5.60	Low	No	No	No	No	No	No	No
Ciprofloxacin	−9.09	High	No	Yes	No	No	No	No	No

BBB, blood-brain barrier; CYP, cytochrome-P; GIA, gastrointestinal absorption; LogKp, skin permeation value; P-gp, P glycoprotein.

**Table 10:** Toxicity analysis of the isolated compounds (**1**–**6**) computed by ProTox-II.

Compound no.	LD_50_ (mg/kg)	Toxicity class	Toxicity				
			Carcino	Cyto	Hepato	Mutagen	Immuno
**1**	890	4	No	No	No	No	Yes
**2**	5000	5	No	No	No	No	Yes
**3**	1050	4	Yes	No	No	No	Yes
**4**	159	3	Yes	No	No	Yes	No
**5**	500	4	No	No	No	No	No
**6**	680	4	No	No	No	No	Yes
Ciprofloxacin	2000	4	No	No	No	Yes	No

Carcino, carcinogenicity; Cyto, cytotoxicity; Hepato, hepatotoxicity; Immuno, immunotoxicity; LD, lethal dose; Mutagen, mutagenicity.

## Data Availability

The NMR data used to support this study are incorporated within the supplementary materials. Additional data are also available from the corresponding author upon request.

## Supplementary Materials

Supplementary Materials The NMR data of the reported compounds are presented as Supplementary materials (Tables S1–6 and Figures S1–31).
