# Supplementary material for: Antibacterial and Antioxidant Efficacies of Secondary Metabolites from the Roots of Cyphostemma adenocaule: A Combined In Vitro and In Silico Study
Source: J Trop Med. 2024 Mar 6;2024:1679695. doi: 10.1155/2024/1679695 (PMC10937085; doi:10.1155/2024/1679695)
Supplement: Supplementary Materials — The NMR data of the reported compounds are presented as Supplementary materials (Tables S1–6 and Figures S1–31). [file 1679695.f1.pdf]

Supplementary Materials

Antibacterial and Antioxidant Efficacy of Secondary Metabolites from the Roots of *Cyphostemma adenocaule*: A Combined *In Vitro* and *In Silico* Study

Hadush Gebrehiwot[1], Yadessa Melaku[1], Muhdin Aliye[1], Urgessa Ensermu[2], Aman Dekebo[1,3], Milkyas Endale[4], Daniel Rentsch[5] and Mo Hunsen[6]

[1]*Department of Applied Chemistry, Adama Science and Technology University, P.O. Box 1888, Adama, Ethiopia*
[2]*Department of Applied Biology, Adama Science and Technology University, P.O. Box 1888, Adama, Ethiopia*
[3]*Institute of Pharmaceutical Sciences, Adama Science and Technology University, P.O. Box 1888, Adama, Ethiopia*
[4]*Traditional and Modern Medicine Research and Development Directorate, Armauer Hansen Research Institute, P.O. Box 1242, Addis Ababa, Ethiopia*
[5]*Laboratory for Functional Polymers, Empa, Swiss Federal Laboratories for Materials Science and Technology, Überlandstrasse 129, 8600 Dübendorf, Switzerland*
[6]*Department of Chemistry, Kenyon College, Gambier, OH 43022, USA*

Correspondence should be addressed to Aman Dekebo; amandekeb@gmail.com

In this file, the tabular and NMR spectral information of all the compounds (**1-6**) used to establish the chemical structures of the isolated compounds from the roots of *C. adenocaule* are depicted in Table S1-6 and Figure S1-31, respectively.

TABLE S1: ^{1}H, ^{13}C, and COSY NMR (400 MHz, $CDCl_3$, δ in ppm, *J* in Hz) spectral data of compound **1**, and ^{1}H and ^{13}C NMR values of *β*-sitosterol in the literature.

C. No.	Compound 1			*β*-Sitosterol [32]	
	^{1}H NMR	^{13}C NMR	COSY	^{1}H NMR	^{13}C NMR
1	1.84 (1H, *m*), 1.07 (1H, *m*)	37.3	H-2	1.83 (1H, *m*), 1.08 (1H, *m*)	37.2
2	1.81 (1H, *m*), 1.52 (1H, *m*)	31.7	H-1	1.82 (1H, *m*), 1.49 (1H, *m*)	32.6
3	3.51 (1H, *m*)	71.9	H-4	3.53 (1H, *m*)	71.8
4	2.27 (1H, *m*), 2.22 (1H, *m*)	42.4	H-3	2.28 (1H, *m*), 2.24 (1H, *m*)	42.3
5	-	140.8		-	140.7
6	5.34 (1H, *m*)	121.8	H-7	5.35 (1H, *d*, *J* = 4.7)	121.7
7	1.98 (1H, *m*), 1.94 (1H, *m*)	32.0	H-6, 8	1.98 (1H, *m*), 1.56 (1H, *m*)	31.9
8	1.49 (1H, *m*)	32.0	H-7	1.46 (1H, *m*)	31.9
9	0.95 (1H, *m*)	50.2		0.94 (1H, *m*)	50.1
10	-	36.6		-	36.5
11	1.45 (1H, *m*), 1.43 (1H, *m*)	21.1		1.48 (1H, *m*), 1.45 (1H, *m*)	21.0
12	2.00 (1H, *m*), 1.13 (1H, *m*)	39.8		1.97 (1H, *m*), 1.15 (1H, *m*)	39.8
13	-	42.3		-	42.2
14	1.03 (1H, *m*)	56.8		1.03 (1H, *m*)	56.7
15	1.55 (1H, *m*), 1.05 (1H, *m*)	24.4		1.55 (1H, *m*), 1.05 (1H, *m*)	24.3
16	1.26 (2H, *m*)	28.3		1.24 (2H, *m*)	28.2
17	1.10 (1H, *m*)	56.1		1.13 (1H, *m*)	56.0
18	0.67 (3H, *s*)	12.0		0.68 (3H, *s*)	11.9
19	1.00 (3H, *s*)	19.5		1.01 (3H, *s*)	19.4
20	1.33 (1H, *m*)	36.2	H-21	1.36 (1H, *m*)	36.1
21	0.91 (3H, *d*, *J* = 6.6)	18.8	H-20	0.94 (3H, *d*)	18.8
22	1.30 (2H, *m*)	34.0		1.32 (2H, *m*)	33.9
23	1.14 (2H, *m*)	26.1		1.15 (2H, *m*)	26.0
24	0.93 (1H, *m*)	45.9		0.93 (1H, *m*)	45.8
25	1.64 (1H, *m*)	29.2	H-26, 27	1.65 (1H, *m*)	29.1
26	0.80 (3H, *d*, *J* = 6.2)	19.1	H-25	0.82 (3H, *d*, *J* = 6.3)	19.0
27	0.81 (3H, *d*, *J* = 6.1)	19.9	H-25	0.83 (3H, *d*, *J* = 6.1)	19.8
28	1.20 (2H, *m*)	23.1	H-29	1.22 (2H, *m*)	23.0
29	0.83 (3H, *t*)	11.9	H-28	0.85 (3H, *t*)	11.8

TABLE S2: ^{1}H, ^{13}C, DEPT-135, COSY, and HMBC NMR (400 MHz, $CDCl_3$, δ in ppm, *J* in Hz) spectral data of compound **2**, and ^{1}H and ^{13}C NMR values of 3-hydroxy-isoagatholactone in the literature.

**C. No.**	**Compound 2**					**3-Hydroxy-isoagatholactone** [33]	
	**^{1}H NMR**	**^{13}C NMR**	**DEPT-135**	**COSY**	**HMBC**	**^{1}H NMR**	**^{13}C NMR**
1	1.94 (1H, *m*), 1.54 (1H, *m*)	32.8	$-CH_2-$			1.60 (2H, *m*)	32.7
2	2.11 (1H, *m*), 1.56 (1H, *m*)	25.1	$-CH_2-$	H-3		1.52 (2H, *m*)	25.0
3	3.42 (1H, *t*, *J* = 2.9)	75.8	-CH-O-	H-2		3.42 (1H, *t*, *J* = 2.9)	75.7
4	-	37.5	Q			-	37.4
5	1.38 (1H, *m*)	54.1	-CH-		C-7, 20	1.36 (1H, *m*)	56.6
6	1.46 (1H, *m*), 1.35 (1H, *m*)	18.0	$-CH_2-$			1.39 (2H, *m*)	17.9
7	1.68 (1H, *m*), 1.66 (1H, *m*)	40.7	$-CH_2-$			1.45 (2H, *m*)	40.6
8	-	34.5	Q			-	34.4
9	1.43 (1H, *m*)	51.1	-CH-	H-11		1.36 (1H, *m*)	51.1
10	-	37.1	Q			-	37.0
11	2.37 (1H, *dd*, *J* = 5.6), 2.32 (1H, *dd*, *J = 5.7*)	24.2	$-CH_2-$	H-9, 12		2.31 (2H, *m*)	24.1
12	6.85 (1H, *dd*, *J* = 3.5, 3.5)	136.4	=CH-	H-11		6.85 (1H, *dd*, *J* = 3.5, 3.5)	136.4
13	-	127.0	Q			-	127.0
14	2.79 (1H, *dd*, *J* = 8.7, 4.4)	49.3	-CH-	H-15		2.79 (1H, *m*)	53.9
15	4.36 (1H, *t*, *J* = 9.2), 4.03 (1H, *t*, *J* = 9.1)	67.3	$-CH_2-O-$	H-14	C-13, 16	4.36 (1H, *t*, *J* = 9.1), 4.03 (1H, *t*, *J* = 9.1)	67.2
16	-	170.3	Q (-C=O)			-	170.3
17	0.76 (3H, *s*)	14.2	$-CH_3$			0.78 (3H, *s*)	14.1
18	0.92 (3H, *s*)	28.4	$-CH_3$			0.94 (3H, *s*)	28.3
19	0.85 (3H, *s*)	22.2	$-CH_3$			0.97 (3H, *s*)	22.1
20	0.95 (3H, *s*)	15.2	$-CH_3$			0.87 (3H, *s*)	15.1
OH	5.28 (1H, *brs*)	-	-			5.32 (1H, *brs*)	-

TABLE S3: ^{1}H, ^{13}C, DEPT-135, COSY, HSQC, and HMBC NMR (400 MHz, CD_3OD, δ in ppm, *J* in Hz) spectral data of compound **3**, and ^{1}H and ^{13}C NMR values of *ε*-viniferin in the literature.

**C. No.**	**Compound 3**						***ε*-Viniferin** [33, 35]	
	**^{1}H NMR**	**^{13}C NMR**	**DEPT-135**	**COSY**	**HSQC**	**HMBC**	**^{1}H NMR**	**^{13}C NMR**
1	-	133.8	Q				-	133.8
2/6	7.14 (2H, *d*, *J* =8.7)	128.1	=CH-	H-3/5	C-2/6	C-4, 7	7.16 (2H, *d*, *J* = 8.6)	128.2
3/5	6.78 (2H, *d*, *J* = 8.7)	116.4	=CH-	H-2/6	H-3/5	C-2/6	6.79 (2H, *d*, *J* = 8.6)	116.3
4	-	158.5	Q				-	159.4
7	5.37 (1H, *d*, *J* = 6.6)	94.8	-CH-O-	H-8	C-7	C-2, 6, 9	5.39 (1H, *d*, *J* = 6.6)	94.8
8	4.35 (1H, *d*, *J* = 6.6)	58.3	-CH-	H-7	C-8	C-1, 7, 9, 10, 10', 11', 14	4.37 (1H, *d*, *J* = 6.6)	58.2
9	-	147.3	Q				-	147.3
10/14	6.18 (2H, *s*)	107.5	=CH-		C-10/14	C-8, 11, 12, 13	6.19 (2H, *s*)	107.6
11, 13	-	160.0	Q				-	159.9
12	6.21 (1H, *s*)	102.3	=CH-		C-12		6.18 (1H, *s*)	102.2
1'	-	131.2	Q				-	131.1
2'/6'	7.03 (2H, *d*, *J* = 8.8)	128.8	=CH-	H-3'/5'	C-2'/6'	C-4', 7'	7.06 (2H, *d*, *J* = 8.7)	128.8
3'/5'	6.66 (2H, *d*, *J* = 8.8)	116.3	=CH-	H-2'/6'	C-3'/5'		6.67 (2H, *d*, *J* = 8.8)	116.3
4'	-	158.4	Q				-	158.3
7'	6.84 (1H, *d*, *J* = 16.7)	130.3	=CH-	H-8'	C-7'	C-14'	6.84 (1H, *d*, *J* = 16.4)	130.3
8'	6.57 (1H, *d*, *J* = 16.3)	123.6	=CH-	H-7'	C-8'	C-2',6', 9'	6.59 (1H, *d*, *J* = 16.5)	123.6
9'	-	136.8	Q				-	136.8
10'	-	120.0	Q				-	120.0
11'	-	162.7	Q				-	162.7
12'	6.26 (1H, *s*)	96.9	=CH-		C-12'		6.25 (1H, *s*)	96.8
13'	-	159.7	Q				-	159.7
14'	6.64 (1H, *s*)	104.3	=CH-		C-14'		6.63 (1H, *s*)	104.3

TABLE S4: ^{1}H, ^{13}C, DEPT-135, HSQC, and HMBC NMR (400 MHz, CD_3OD, δ in ppm, *J* in Hz) spectral data of compound **4**, and ^{1}H and ^{13}C NMR values of myricetin in the literature.

**C. No.**	**Compound 4**					**Myricetin** [37]	
	**^{1}H NMR**	**^{13}C NMR**	**DEPT-135**	**HSQC**	**HMBC**	**^{1}H NMR**	**^{13}C NMR**
1	-	-	-			-	-
2	-	147.9	Q			-	148.0
3	-	137.3	Q			-	137.0
4	-	177.2	Q (–C=O)			-	177.3
5	-	162.4	Q			-	162.5
6	6.18 (1H, *d*, *J* = 2.0)	99.2	=CH-	C-6	C-8, 10	6.17 (1H, *d*, *J* = 2.1)	99.3
7	-	165.5	Q			-	165.6
8	6.38 (1H, *d*, *J* = 2.0)	94.3	=CH-	C-8	C-6, 10	6.37 (1H, *d*, *J* =2.1)	94.4
9	-	158.1	Q			-	158.2
10	-	104.4	Q			-	104.5
1'	-	123.0	Q			-	123.2
2'	7.35 (1H, *s*)	108.5	=CH-	C-2'	C-1', 2, 3', 4', 6',	7.34 (1H, *s*)	108.6
3'	-	146.7	Q			-	146.8
4'	-	136.9	Q			-	137.4
5'	-	146.7	Q			-	146.8
6'	7.35 (1H, *s*)	108.5	=CH-	C-6'	C-1', 2, 2' 4', 5'	7.34 (1H, *s*)	108.6

- ✓ The spectra have some diethyl phthalate contaminant at δ_H 7.71, 7.60, 4.33 and 1.34, and δ_C 169.2, 132.3, 129.8, 62.8 and 14.3.

TABLE S5: ^{1}H, ^{13}C, DEPT-135, COSY, HSQC, and HMBC NMR (400 MHz, CD_3OD, δ in ppm, *J* in Hz) spectral data of compound **5**, and ^{1}H and ^{13}C NMR values of tricuspidatol A in the literature.

C. No.	**Compound 5**						**Tricuspidatol A** [33, 38]	
	**^{1}H NMR**	**^{13}C NMR**	**DEPT-135**	**COSY**	**HSQC**	**HMBC**	**^{1}H NMR**	**^{13}C NMR**
1,1'	-	133.8	Q				-	133.9
2/2'	7.28 (2H, *d*, *J* = 8.6)	128.8	=CH-	H-3 /3'	C-2/2'	C-4, 6, 7/4', 6', 7'	7.27 (2H, *d*, *J* = 8.4)	128.8
3/3'	6.77 (2H, *d*, *J* = 8.6)	116.0	=CH-	H-2 /2'	C-3/3'	C-1, 4, /1', 4'	6.76 (2H, *d*, *J* = 8.4)	116.1
4, 4'	-	157.9	Q				-	158.0
5/5'	6.77 (2H, *d*, *J* = 8.6)	116.0	=CH-	H-6 /6'	C-5/5'	C-1, 4, /1', 4'	6.76 (2H, *d*, *J* = 8.4)	116.1
6/6'	7.28 (2H, *d*, *J* = 8.6)	128.8	=CH-	H-5 /5'	C-6/6'	C-1, 4, 7/1', 4', 7'	7.27 (2H, *d*, *J* = 8.4)	128.8
7/7'	5.41 (2H, *d*, *J* = 6.5)	85.9	-CH-O-	H-8 /8'	C-7/7'		5.40 (2H, *d*, *J* = 4.4)	85.9
8/8'	3.59 (2H, *d*, *J* = 6.7)	59.0	-CH-	H-7 /7'	C-8/8'	C-10, 14, /10', 14'	3.58 (2H, *d*, *J* = 4.4)	59.0
9, 9'	-	142.1	Q				-	142.1
10/10'	6.03 (2H, *s*)	108.9	=CH-		C-10/10'	C-8, 12, /8', 12'	6.02 (2H, *s*)	109.0
11, 11'	-	158.7	Q				-	158.8
12/12'	5.98 (2H, *s*)	101.8	=CH-		C-12/12'		6.23 (2H, *s*)	101.8
13, 13'	-	158.7	Q				-	158.8
14/14'	6.03 (2H, *s*)	108.9	=CH-		C-14/14'	C-8, 12, /8', 12'	6.02 (2H, *s*)	109.0

TABLE S6: ^{1}H, ^{13}C, DEPT-135, COSY, HSQC, and HMBC NMR (400 MHz, CD_3OD, δ in ppm, J in Hz) spectral data of compound **6** and ^{1}H and ^{13}C NMR values of parthenocissin A in the literature.

**C. No.**	**Compound 6**						**Parthenocissin A** [39]	
	**^{1}H NMR**	**^{13}C NMR**	**DEPT-135**	**COSY**	**HSQC**	**HMBC**	**^{1}H NMR**	**^{13}C NMR**
1	-	130.5	Q				-	129.9
2/6	7.19 (2H, *d*, *J* = 8.5)	130.8	=CH-	H-3/5	C-2/6	C-1, 4, 7	7.20 (2H, *d*, *J* = 8.3)	130.6
3/5	6.78 (2H, *d*, *J* = 8.5)	116.1	=CH-	H-2/6	C-3/5	C-2/6	6.72 (2H, *d*, *J* = 8.3)	115.8
4	-	157.4	Q				-	157.1
7	6.25 (1H, *s*)	125.6	=CH-		C-7	C-2/6	6.31 (1H, *s*)	125.2
8	-	150.2	Q				-	149.8
9	-	143.4	Q				-	142.8
10	-	128.3	Q				-	128.0
11	-	155.6	Q				-	155.4
12	6.18 (1H, *s*)	104.2	=CH-		C-12		6.26 (1H, *d*, *J* = 1.5)	104.1
13	-	158.4	Q				-	158.4
14	6.45 (1H, *s*)	103.6	=CH-		C-14	C-12	6.52 (1H, *d*, *J* = 1.5)	103.1
1'	-	138.1	Q				-	137.4
2'/6'	6.94 (2H, *d*, *J* = 8.5)	129.1	=CH-	H-3'/5'	C-2'/6'	C-4', 7'	6.99 (2H, *d*, *J* = 8.8)	128.9
3'/5'	6.69 (2H, *d*, *J* = 8.6)	115.9	=CH-	H-2'/6'	C-3'/5'	C-1'	6.80 (2H, *d*, *J* = 8.8)	115.8
4'	-	156.4	Q				-	156.4
7'	4.19 (1H, *d*, *J* = 2.7)	55.6	-CH-	H-8'	C-7'	C-1', 9'	4.26 (1H, *d*, *J* = 1.8)	54.9
8'	3.68 (1H, *d*, *J* = 2.2)	65.1	-CH-	H-7'	C-8'	C-9', 10',14'	3.45 (1H, *brs*)	64.4
9'	-	146.2	Q				-	145.6
10'/14'	6.14 (2H, *s*)	106.9	=CH-		C-10'/14'		6.19 (2H, *s*)	106.9
11', 13'	-	159.3	Q				-	159.3
12'	6.11 (1H, *s*)	101.5	=CH-		C-12'		6.19 (1H, *s*)	101.4

- ✓ The spectra also showed an acrylate contaminant at δ_H 6.04, 6.00, 5.49, and 1.90, and at δ_C 175.2, 136.7, 125.0 and 24.2. Preparative TLCs are made up of silica gel, and to adhere it to the glass, some manufacturers use acrylates as an adhering agent. There is a likely probability that these adhering agents were detected by NMR considering the fact that the final purification in our case was done by PTLC. During filtration acetone and methanol solvents were used, and acetone likely broke down the acrylic polymer used as a binding agent and possibly the acrylates mixed with the filtrate.

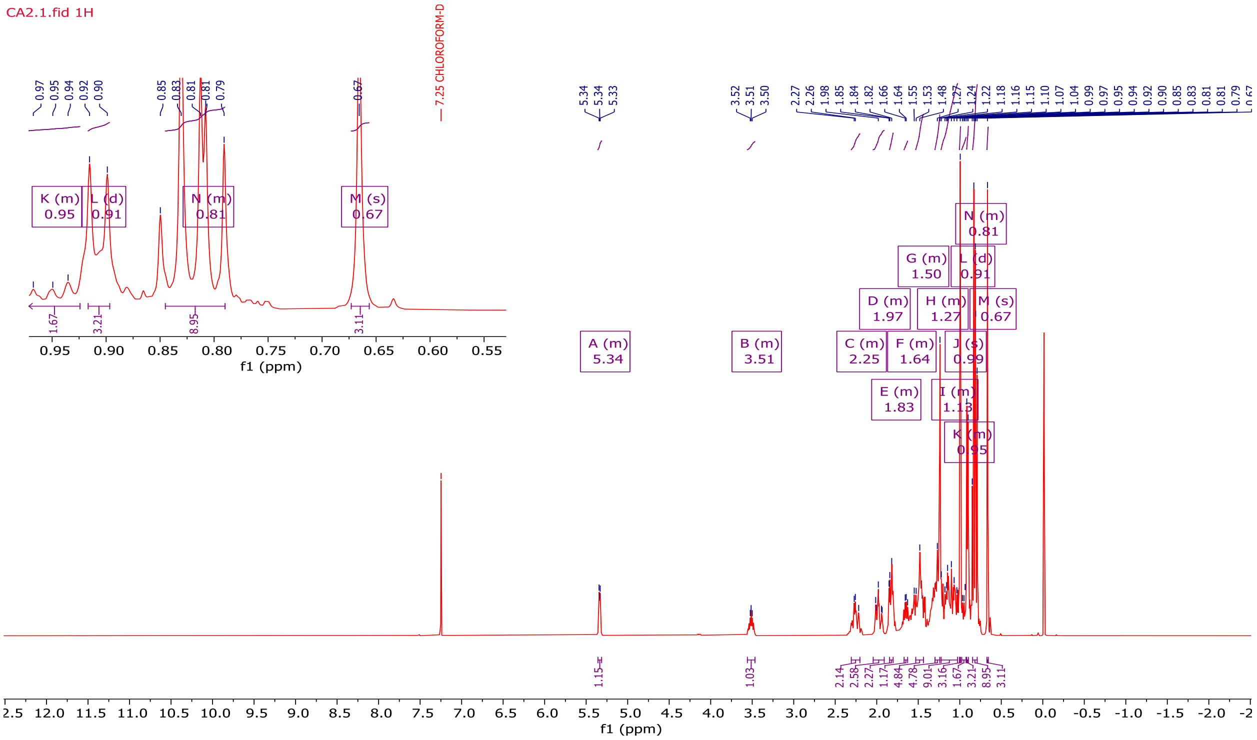

FIGURE S1: ^{1}H NMR spectrum of compound **1**.

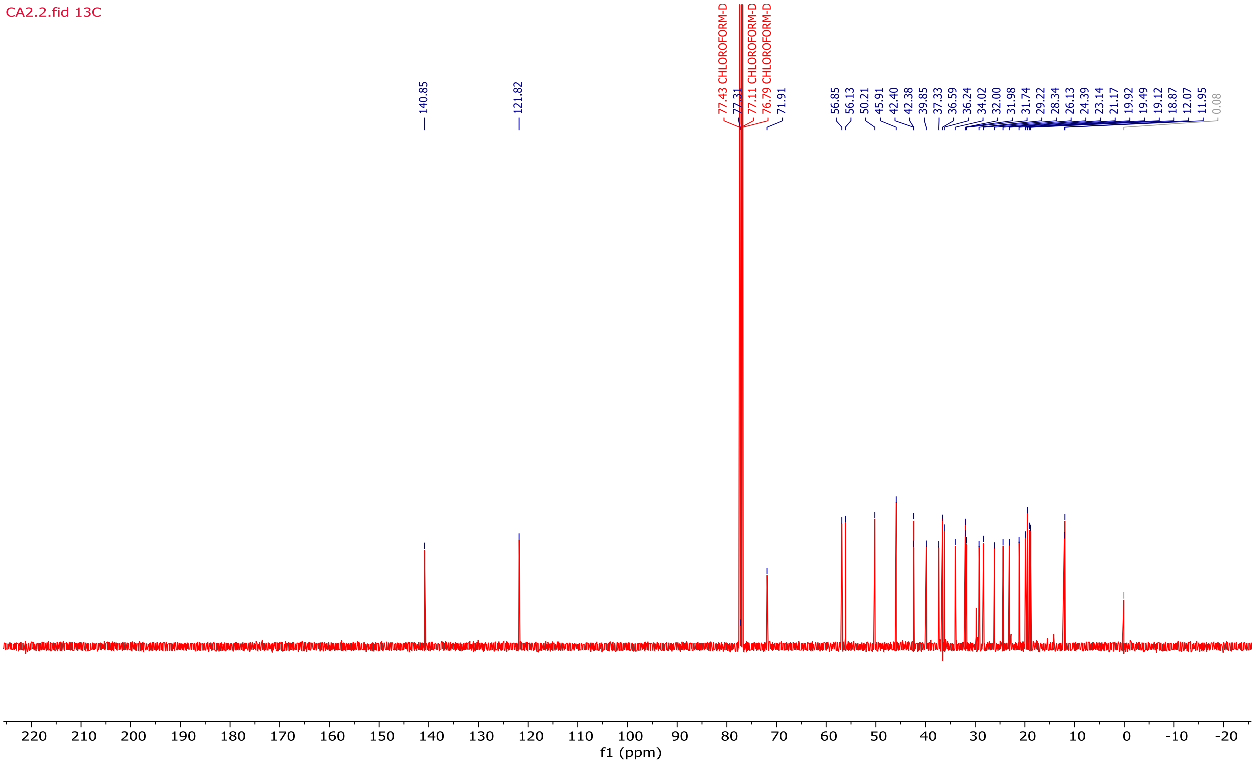

FIGURE S2: ^{13}C NMR spectrum of compound **1**.

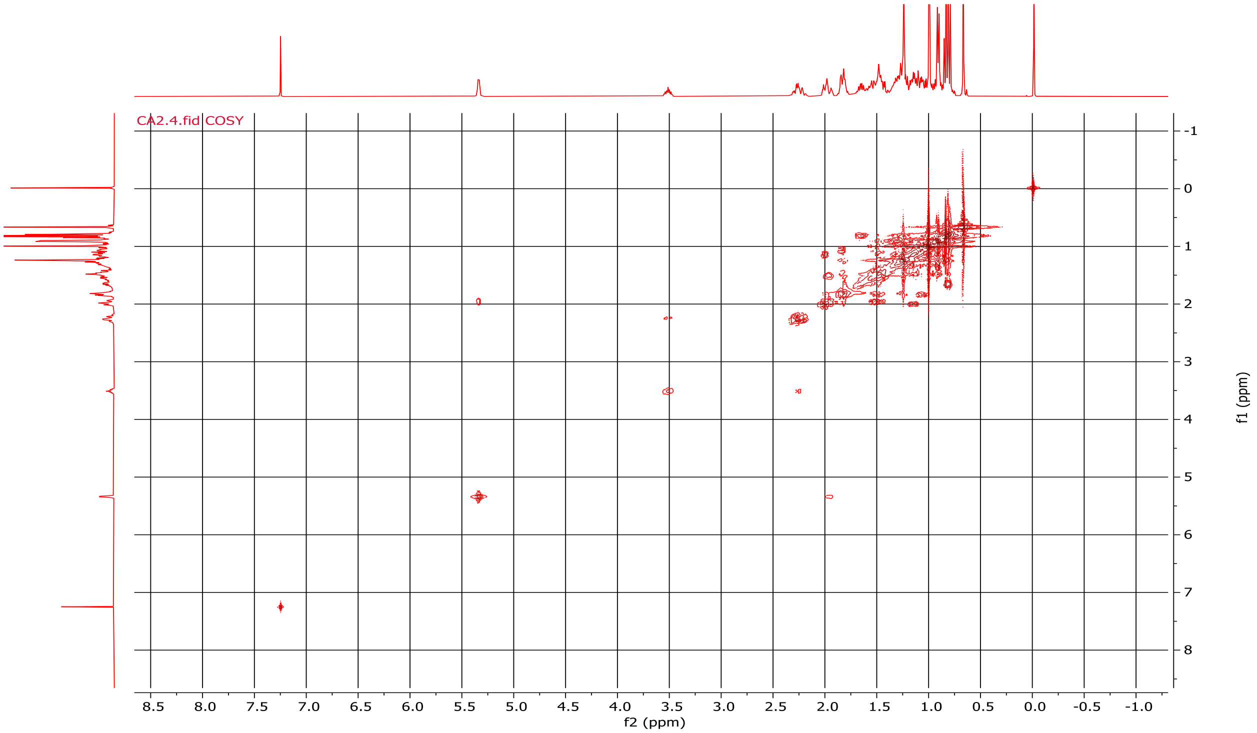

FIGURE S3: COSY spectrum of compound **1**.

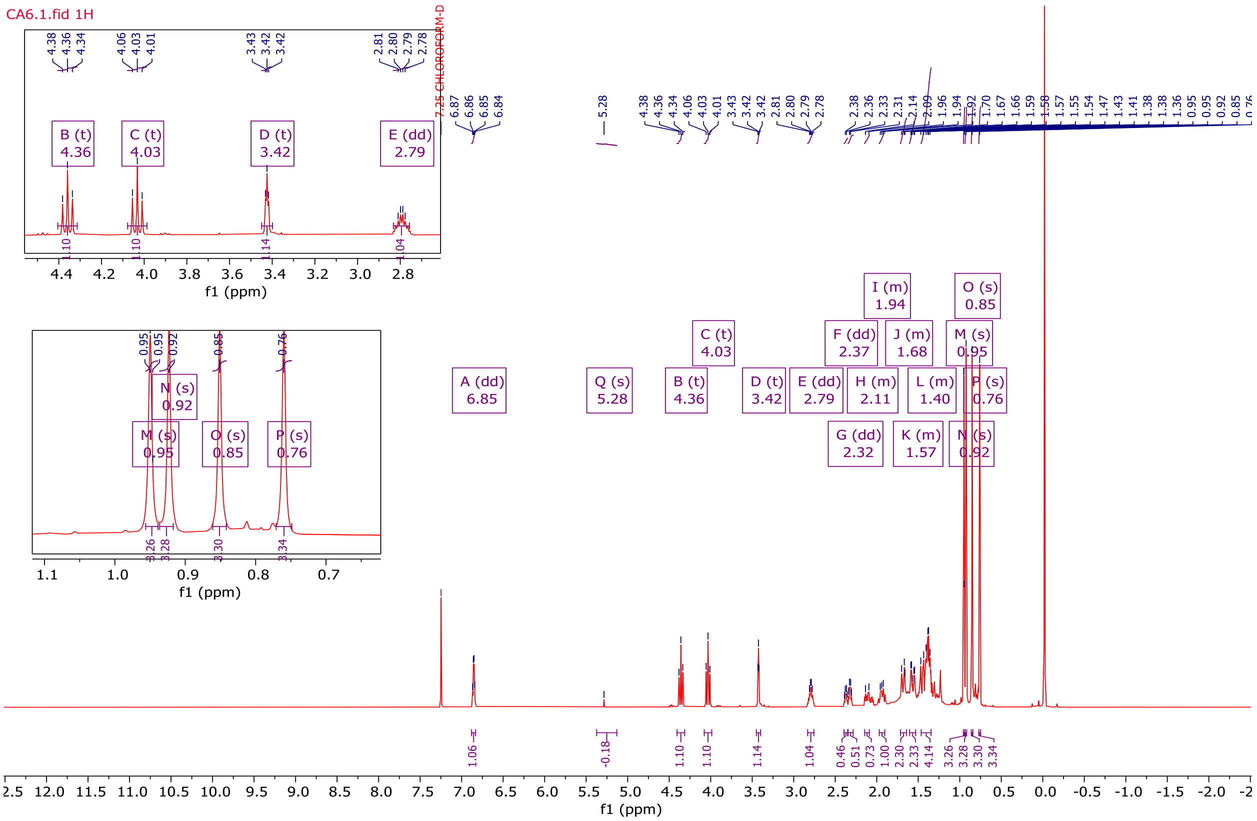

FIGURE S4: ^{1}H NMR spectrum of compound **2**.

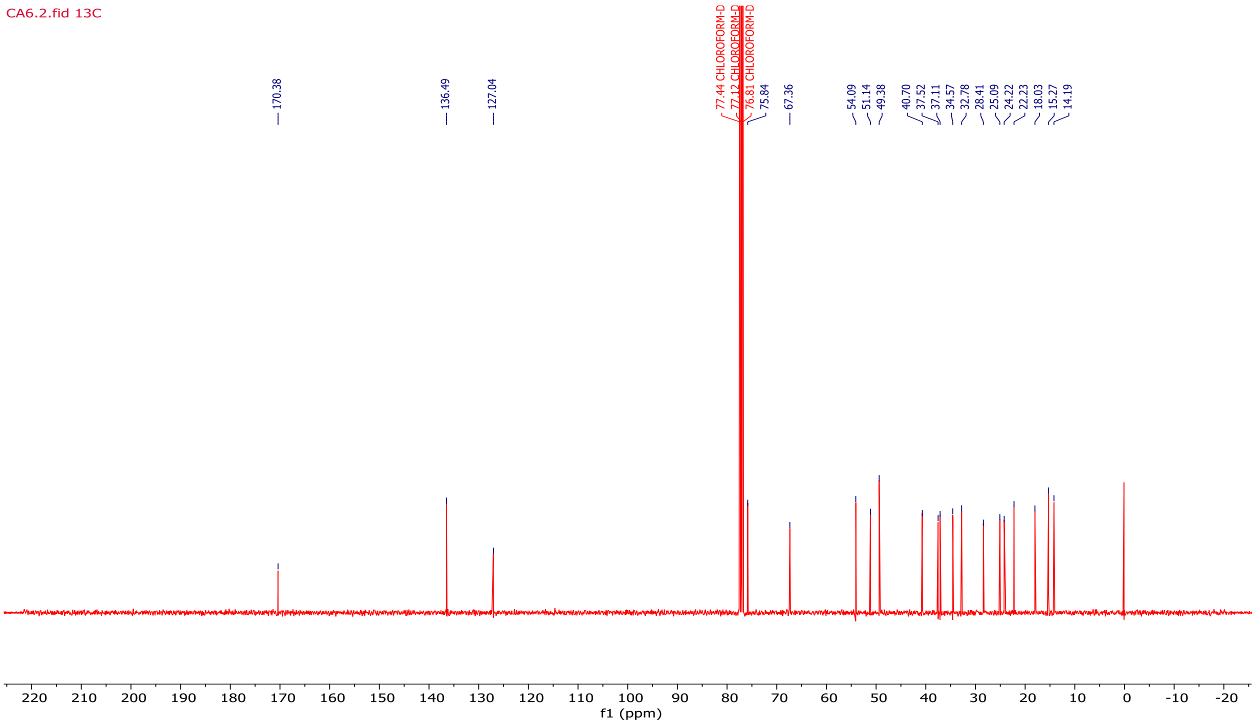

FIGURE S5: ^{13}C NMR spectrum of compound **2**.

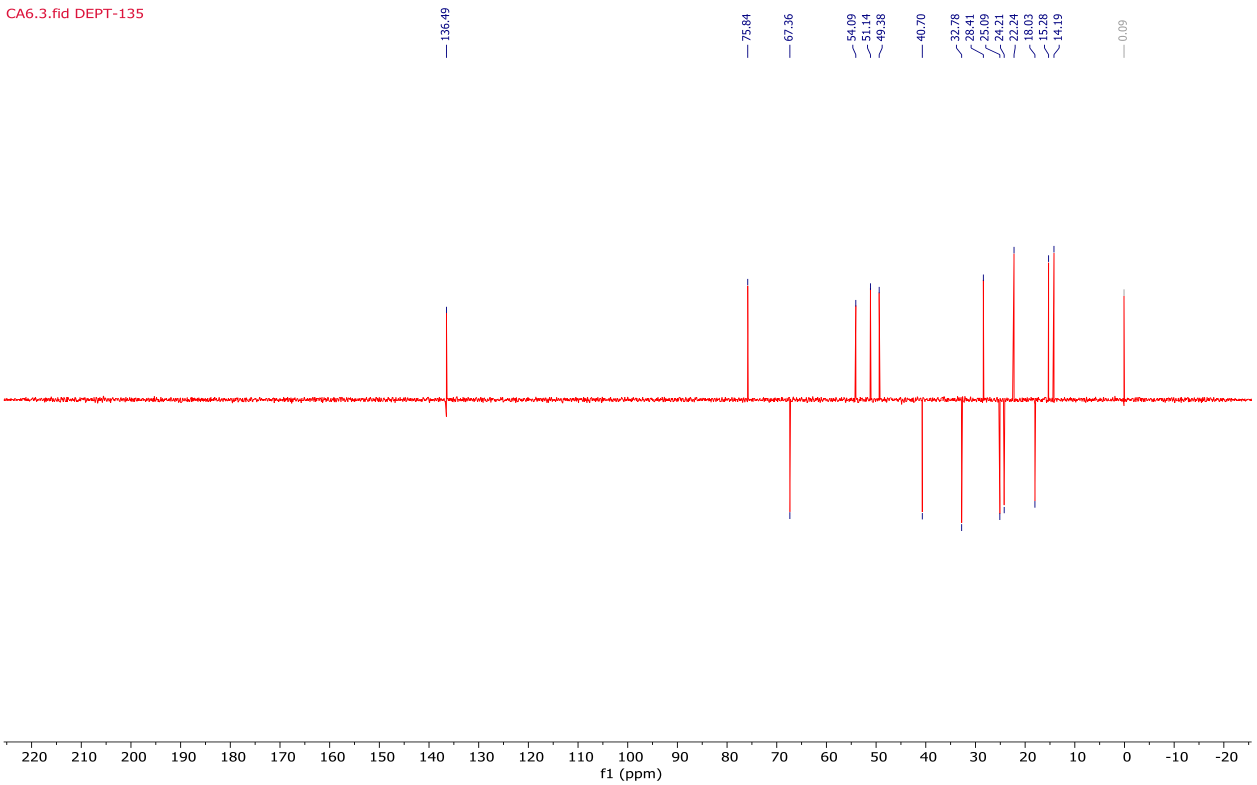

FIGURE S6: DEPT-135 spectrum of compound **2**.

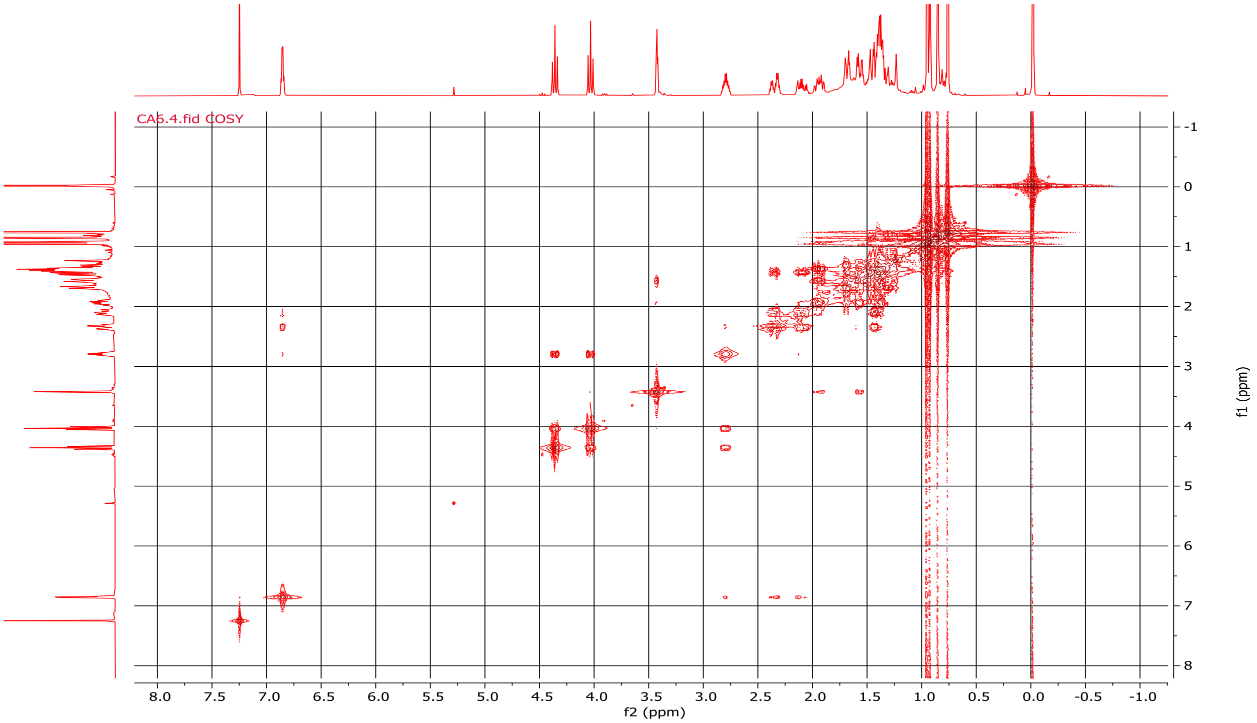

FIGURE S7: COSY spectrum of compound **2**.

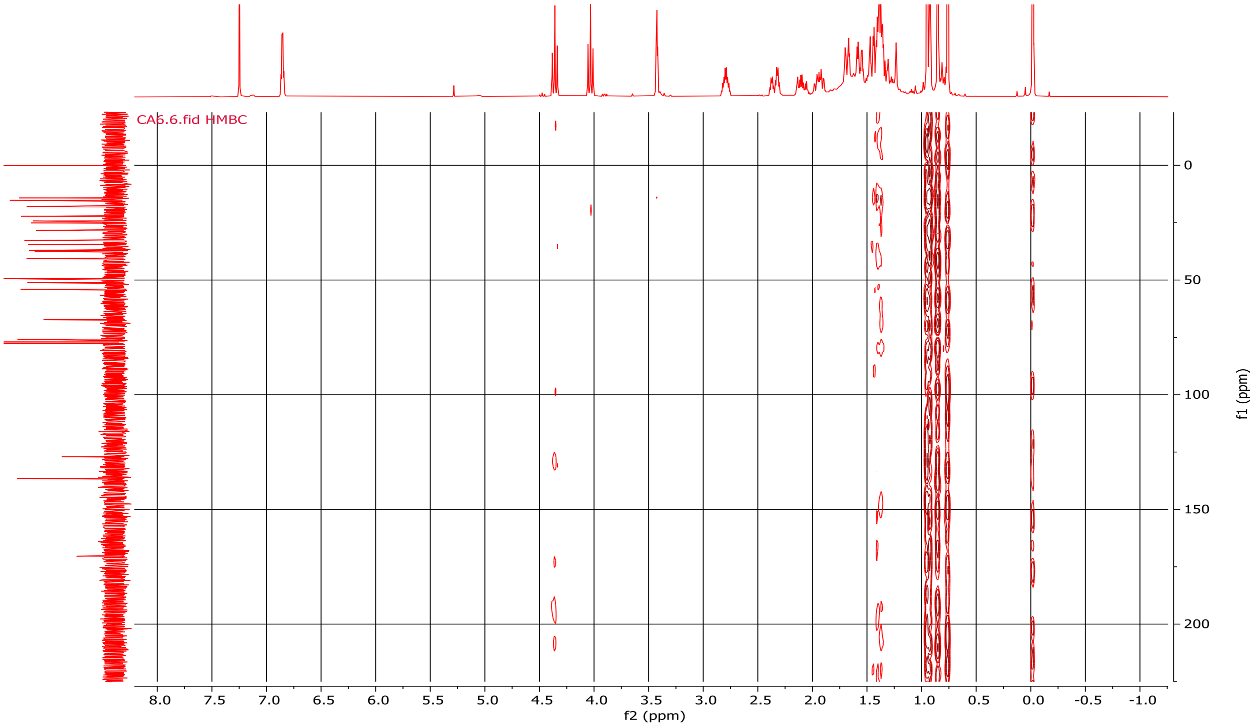

FIGURE S8: HMBC spectrum of compound **2**.

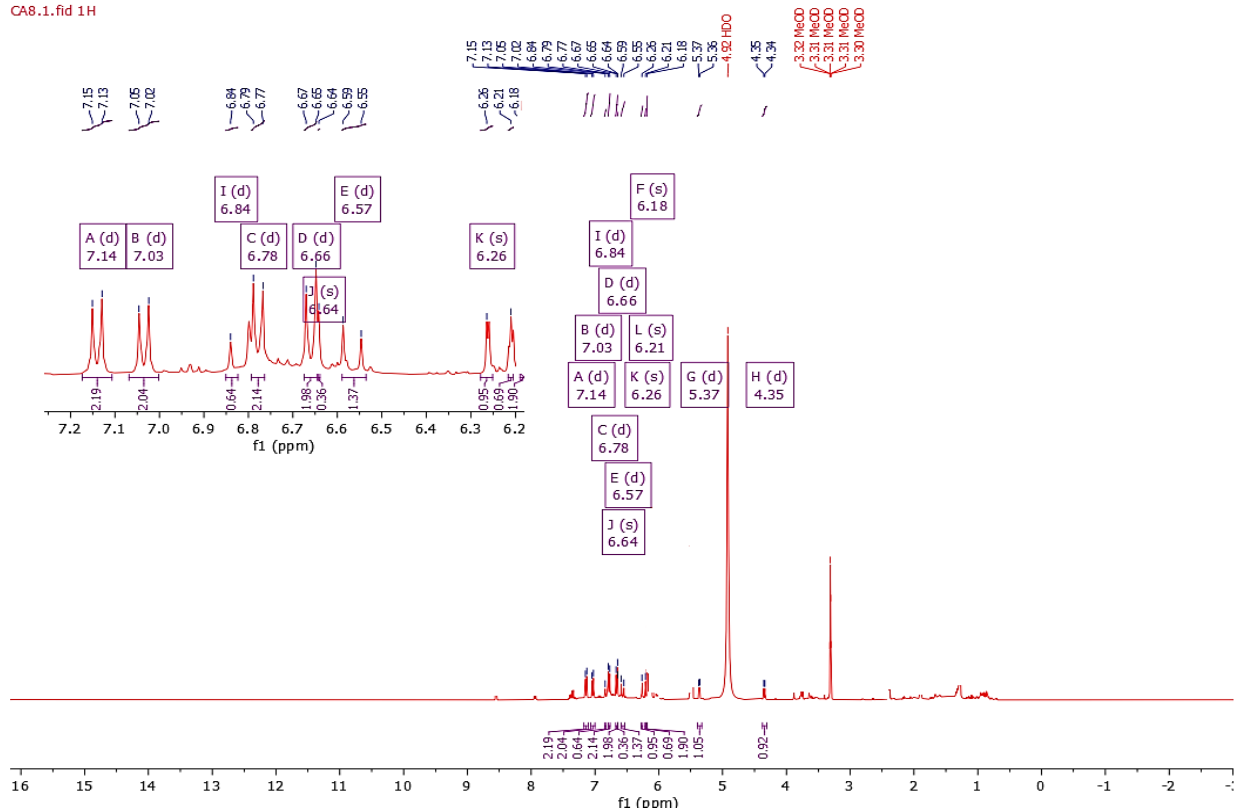

FIGURE S9: ^{1}H NMR spectrum of compound **3**.

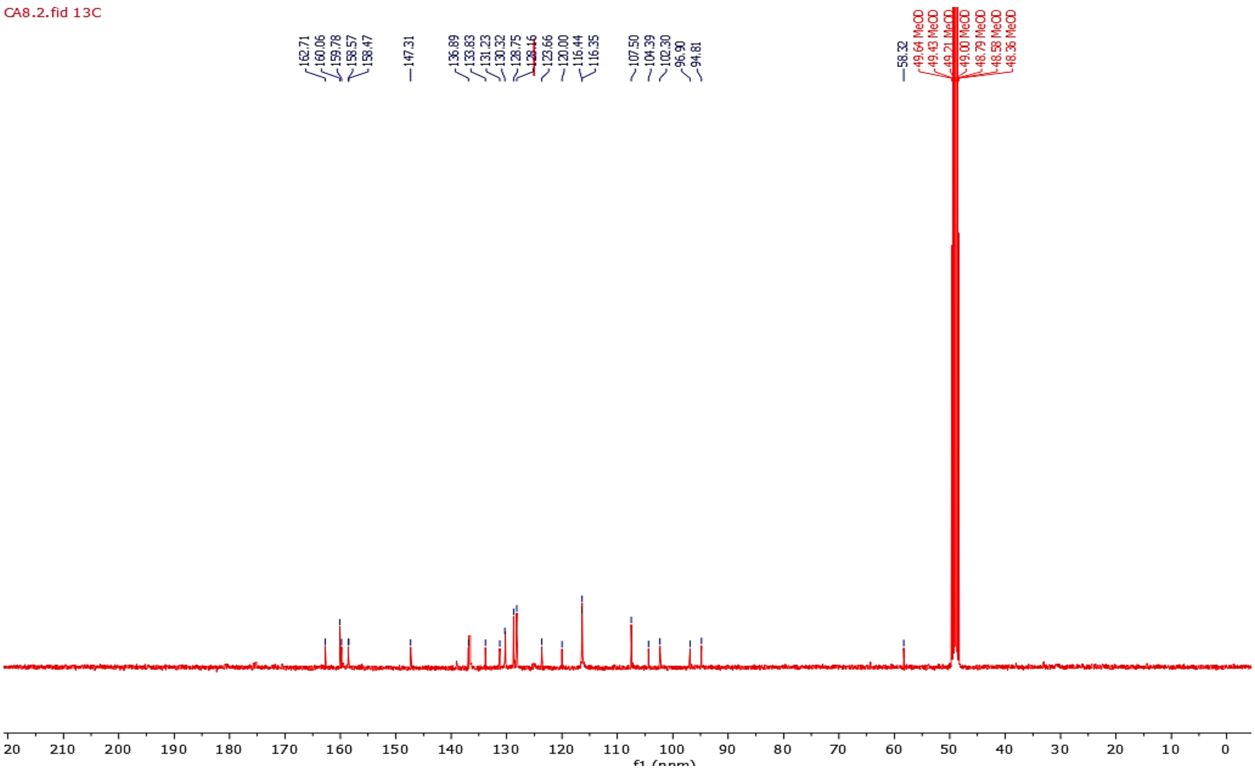

FIGURE S10: ^{13}C NMR spectrum of compound **3**.

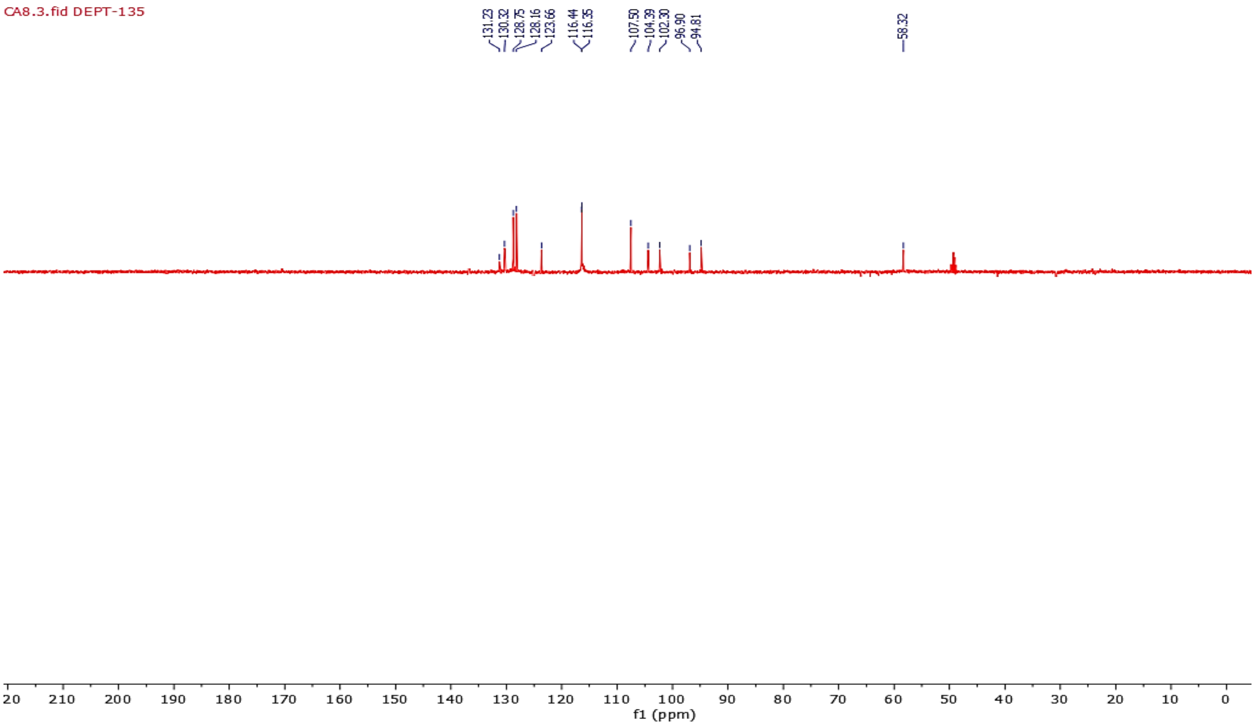

FIGURE S11: DEPT-135 spectrum of compound **3**.

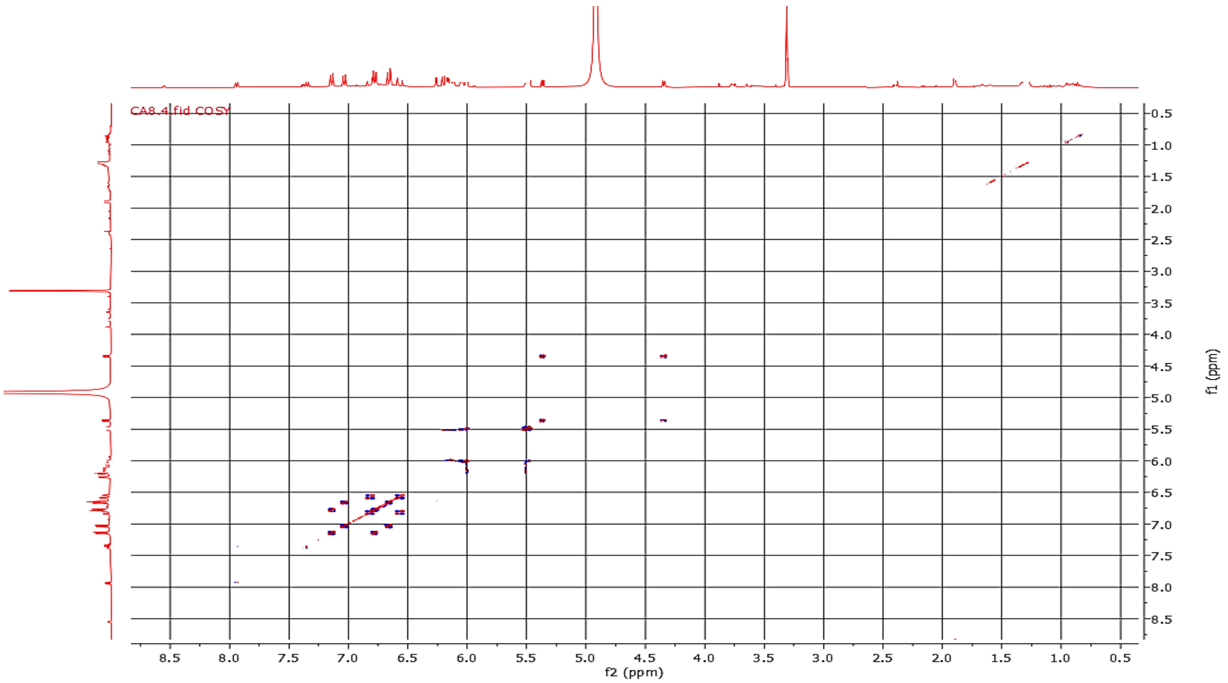

FIGURE S12: COSY spectrum of compound **3**.

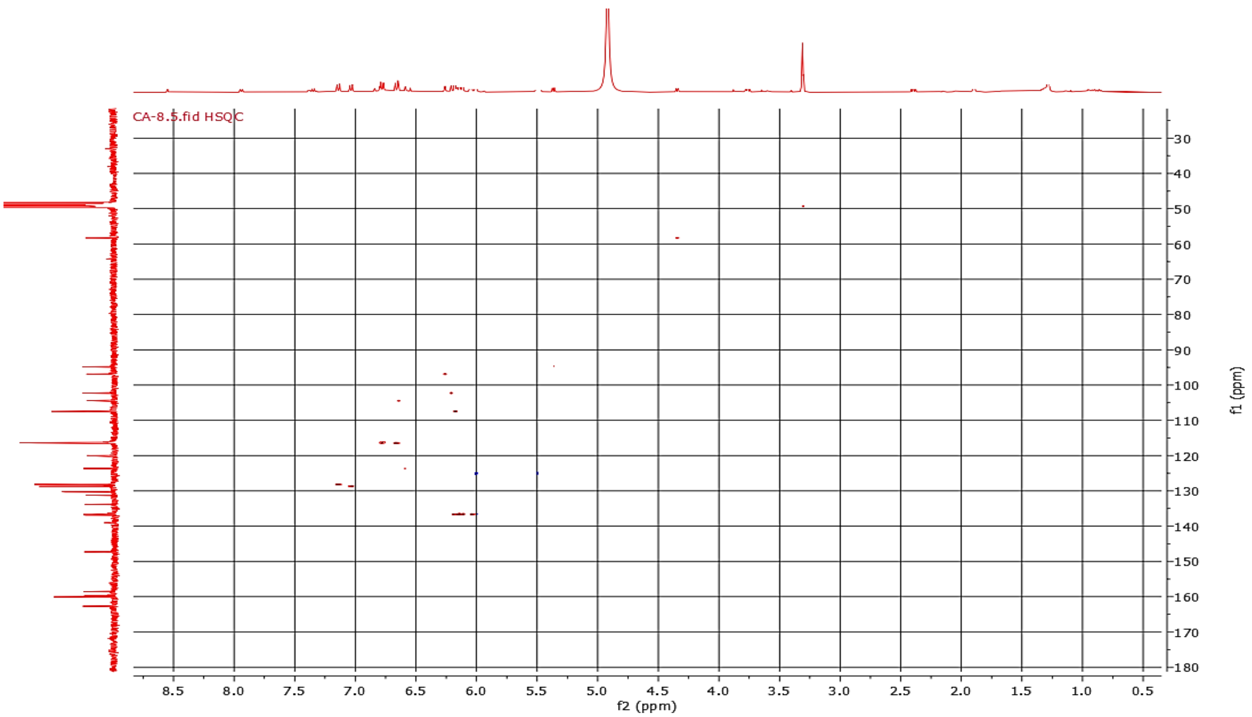

FIGURE S13: HSQC spectrum of compound **3**.

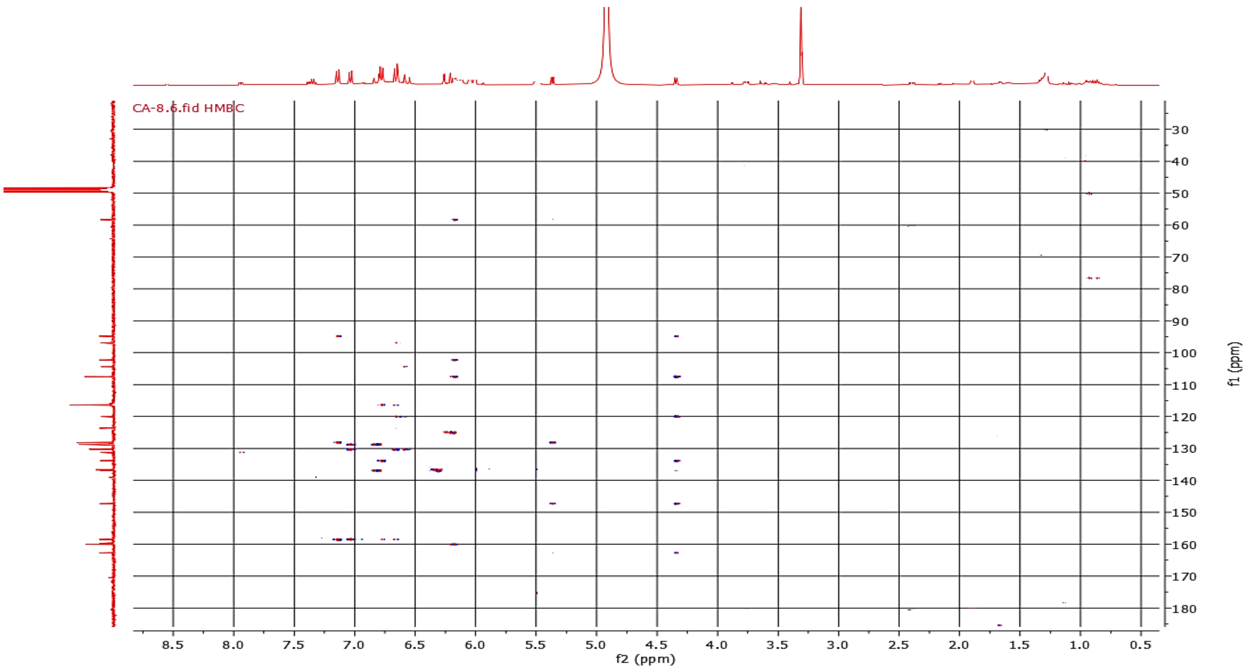

FIGURE S14: HMBC spectrum of compound **3**.

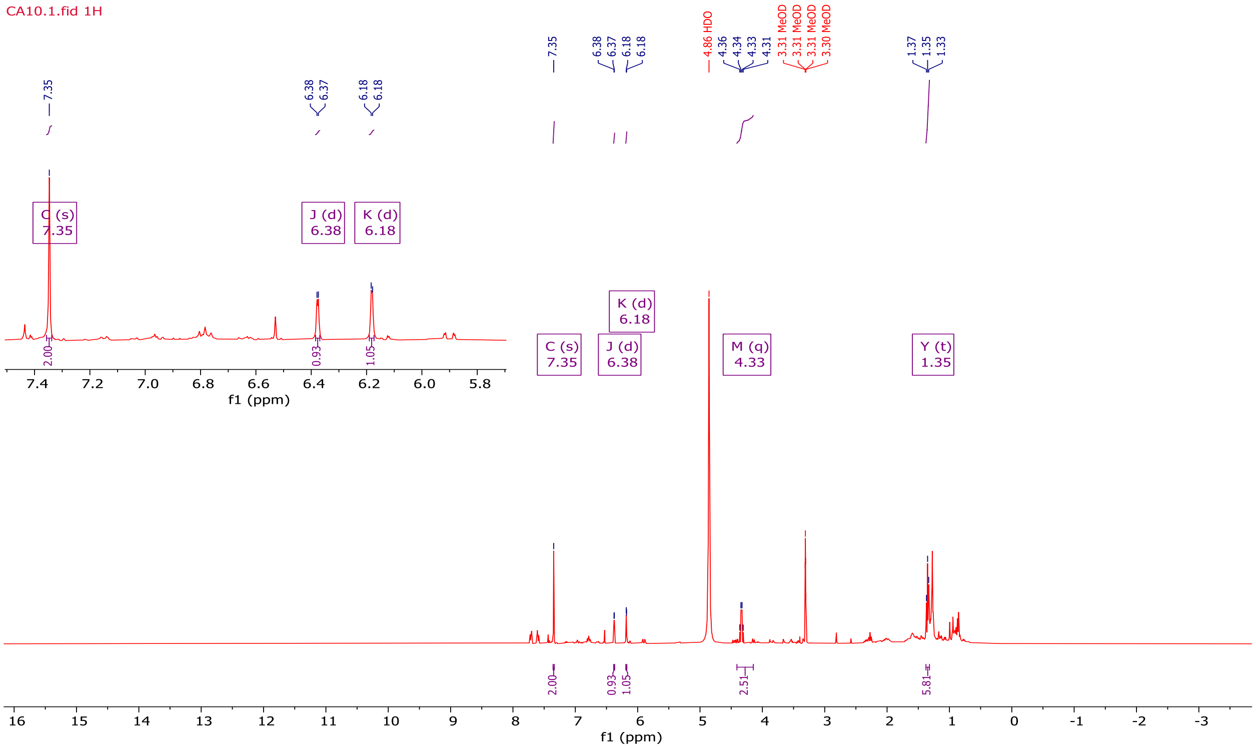

FIGURE S15: ^{1}H NMR spectrum of compound **4**.

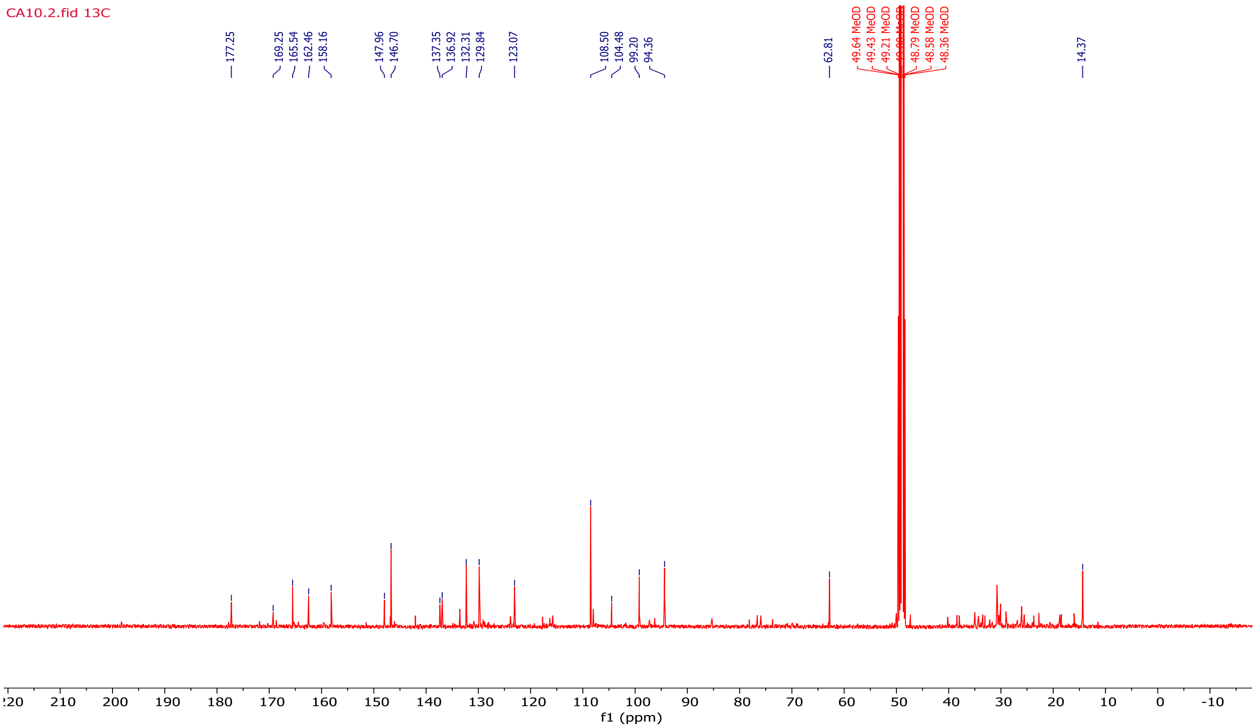

FIGURE S16: ^{13}C NMR spectrum of compound **4**.

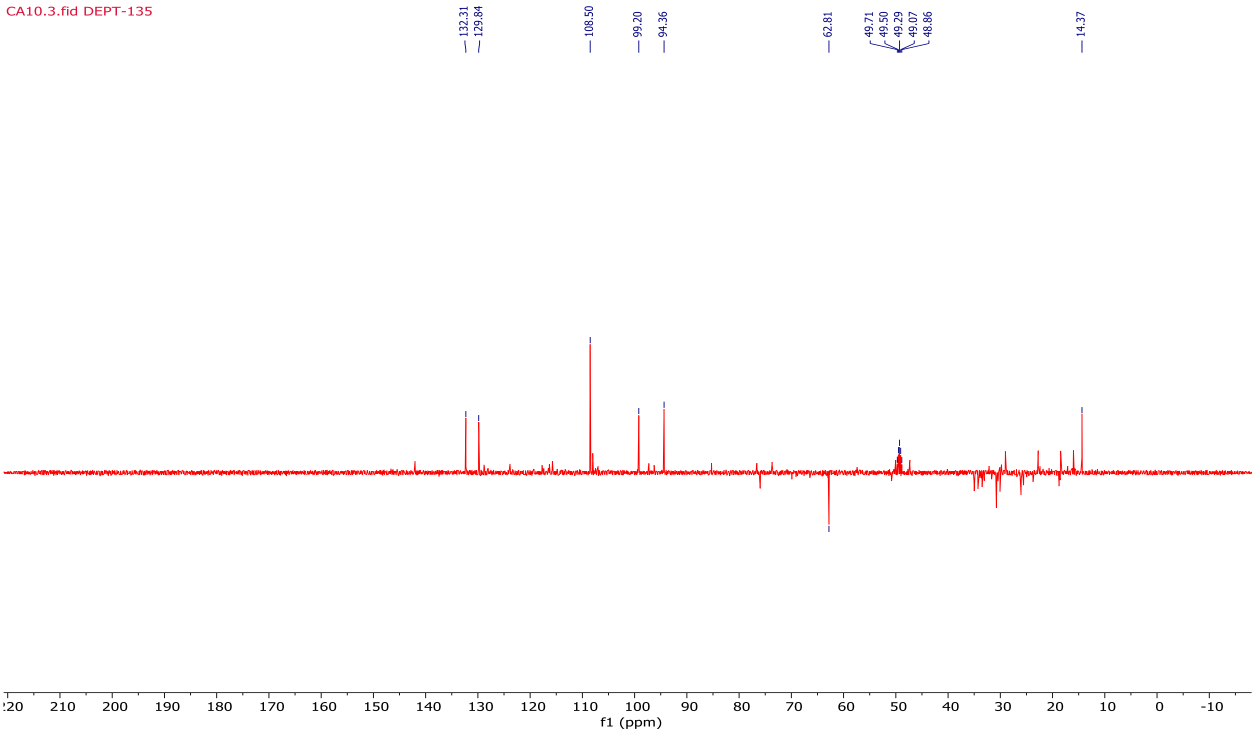

FIGURE S17: DEPT-135 spectrum of compound **4**.

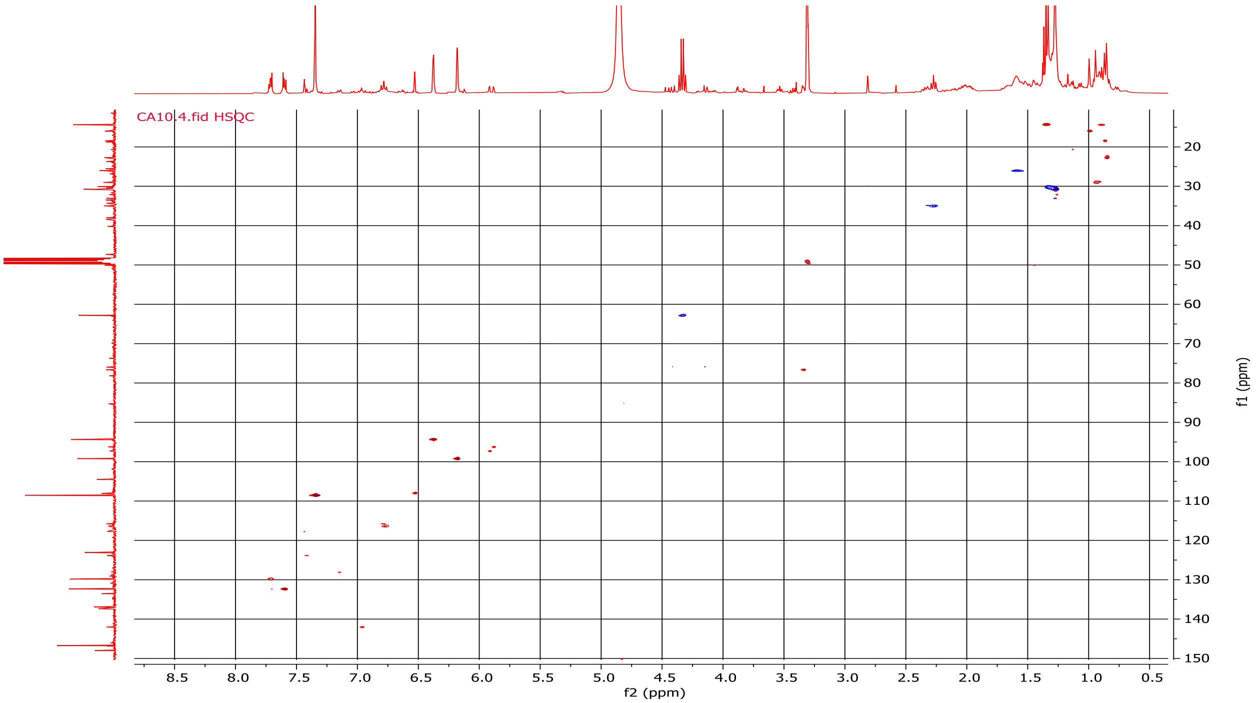

FIGURE S18: HSQC spectrum of compound **4**.

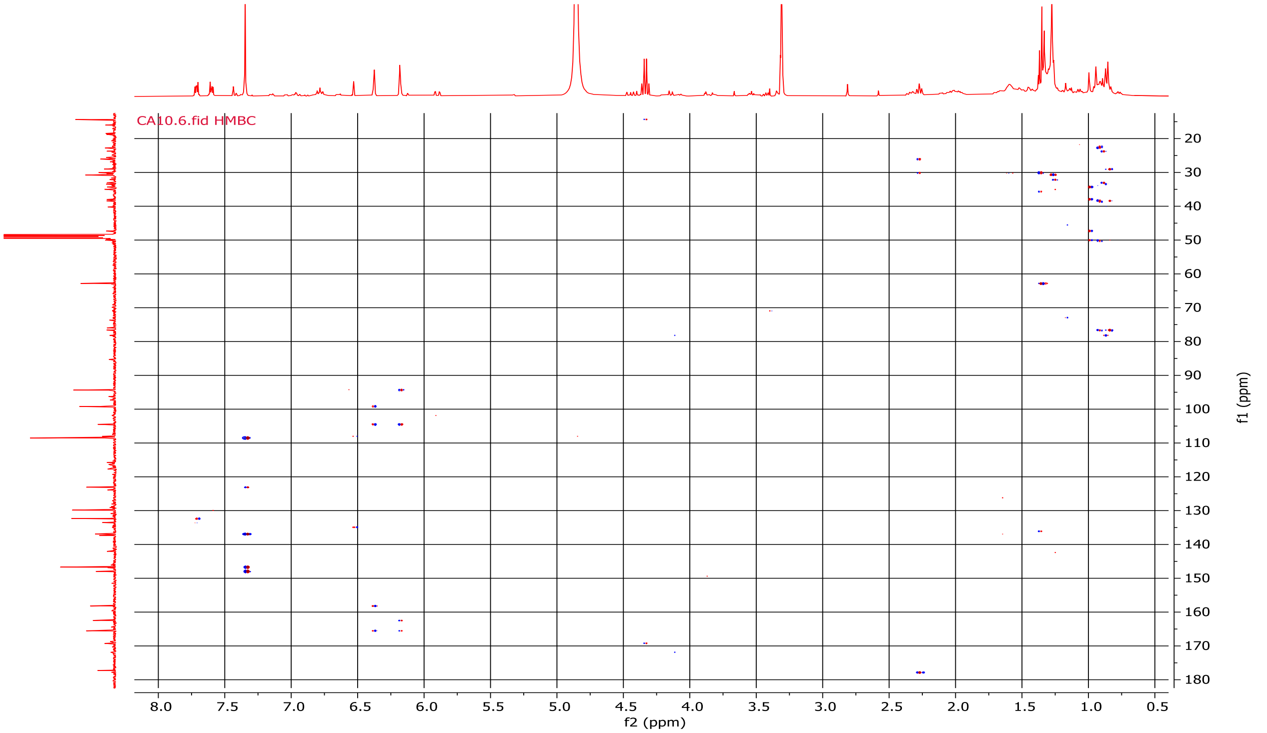

FIGURE S19: HMBC spectrum of compound **4**.

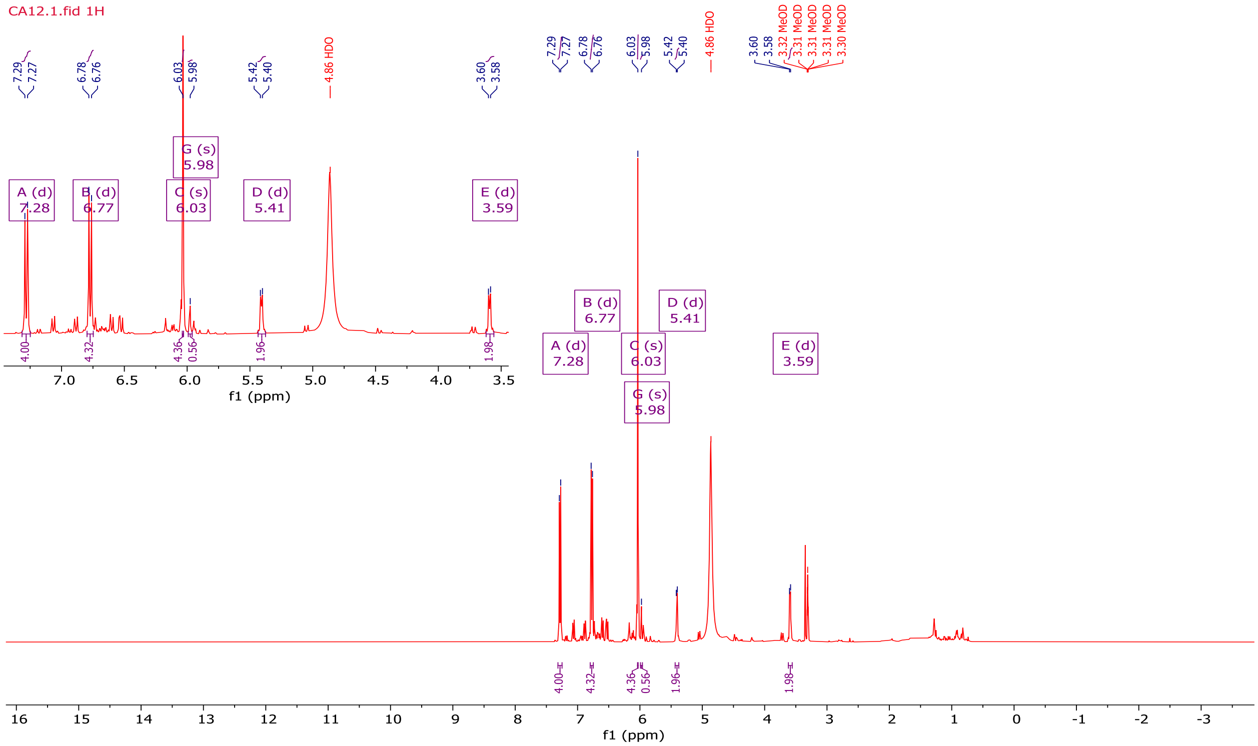

FIGURE S20: ^{1}H NMR spectrum of compound **5**.

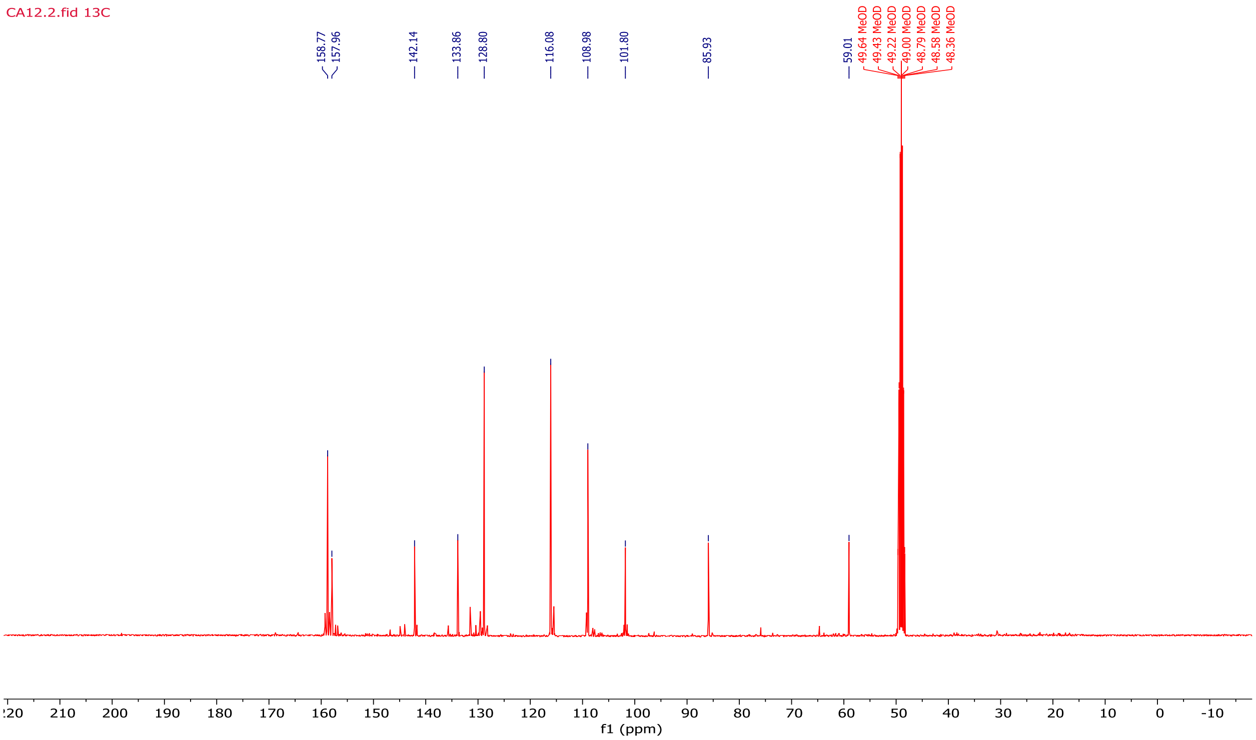

FIGURE S21: ^{13}C NMR spectrum of compound **5**.

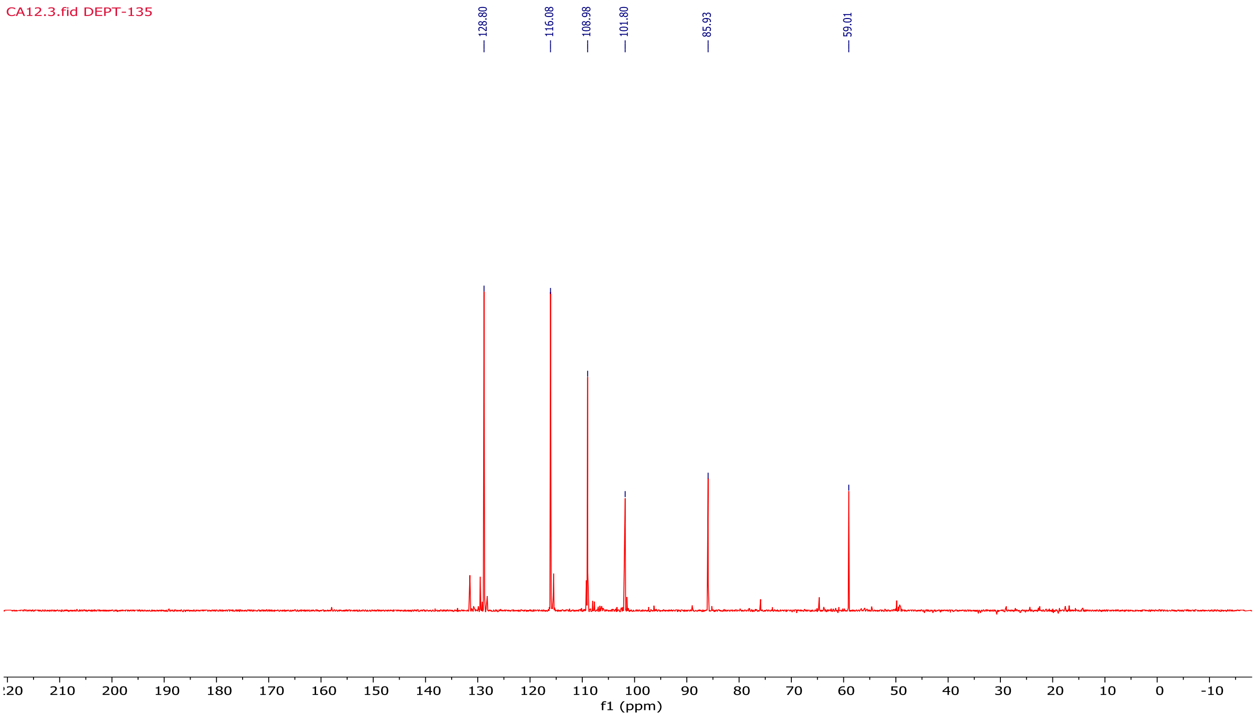

FIGURE S22: DEPT-135 spectrum of compound **5**.

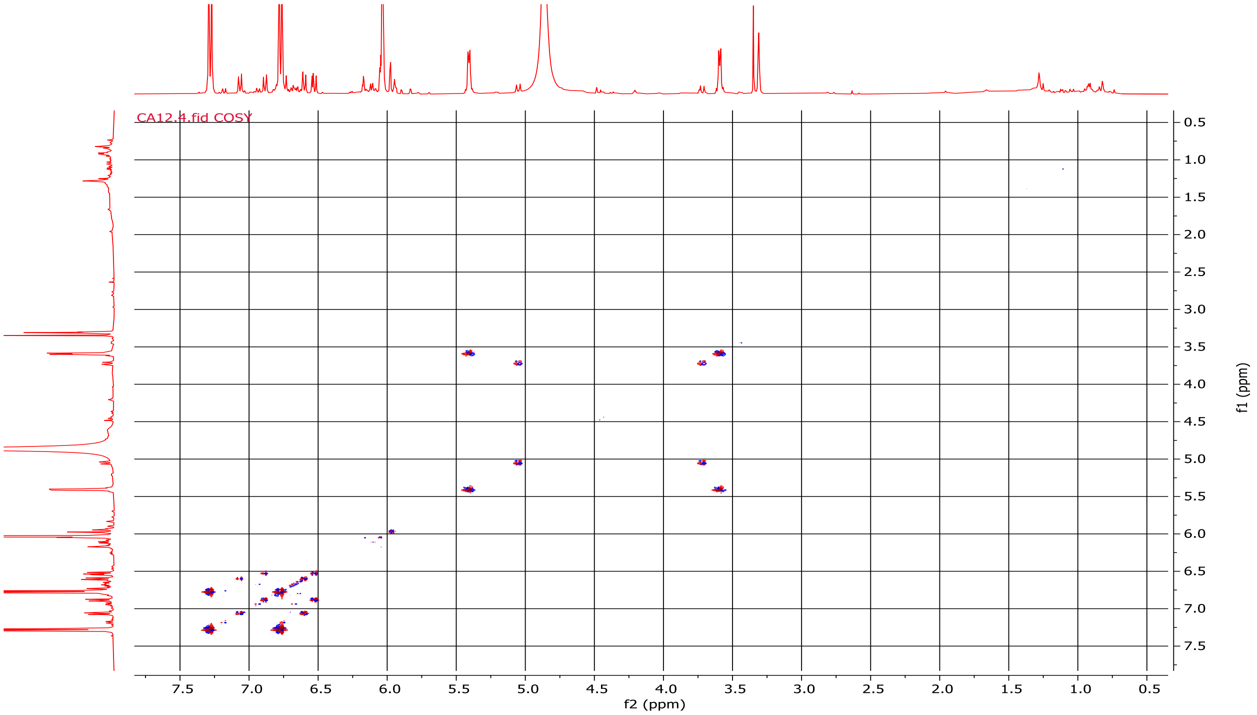

FIGURE S23: COSY spectrum of compound **5**.

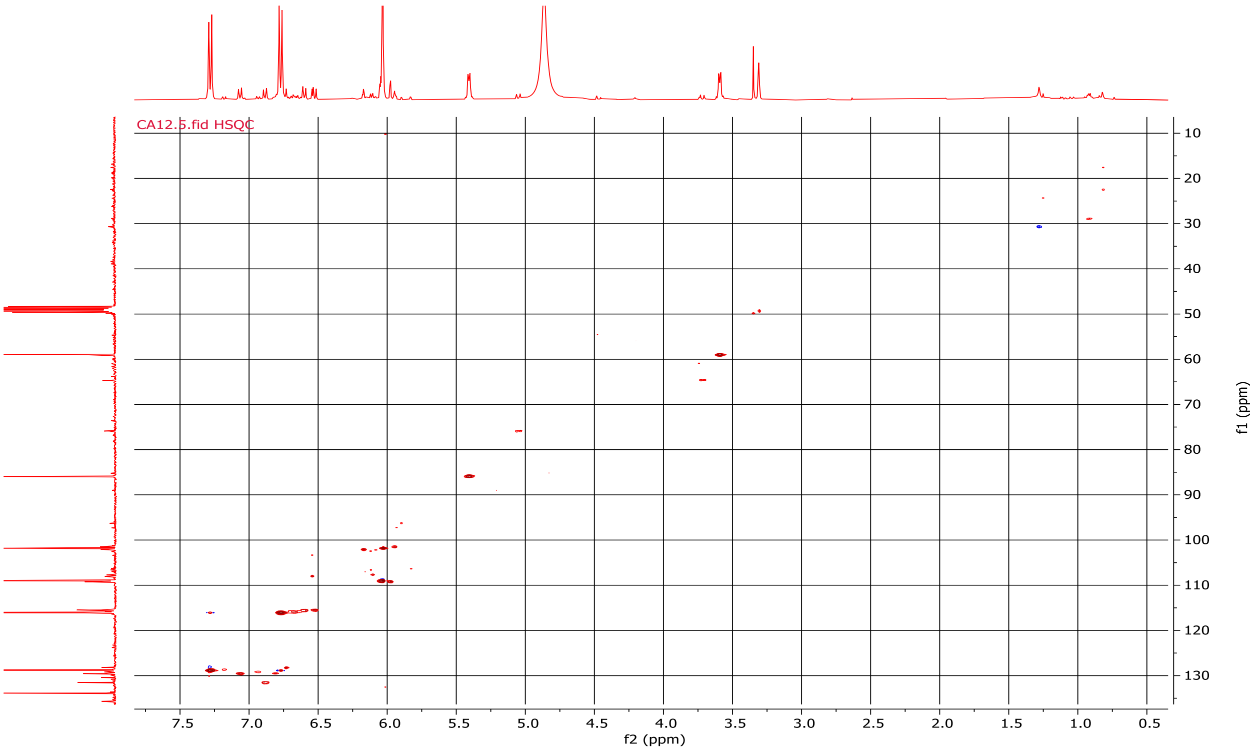

FIGURE S24: HSQC spectrum of compound **5**.

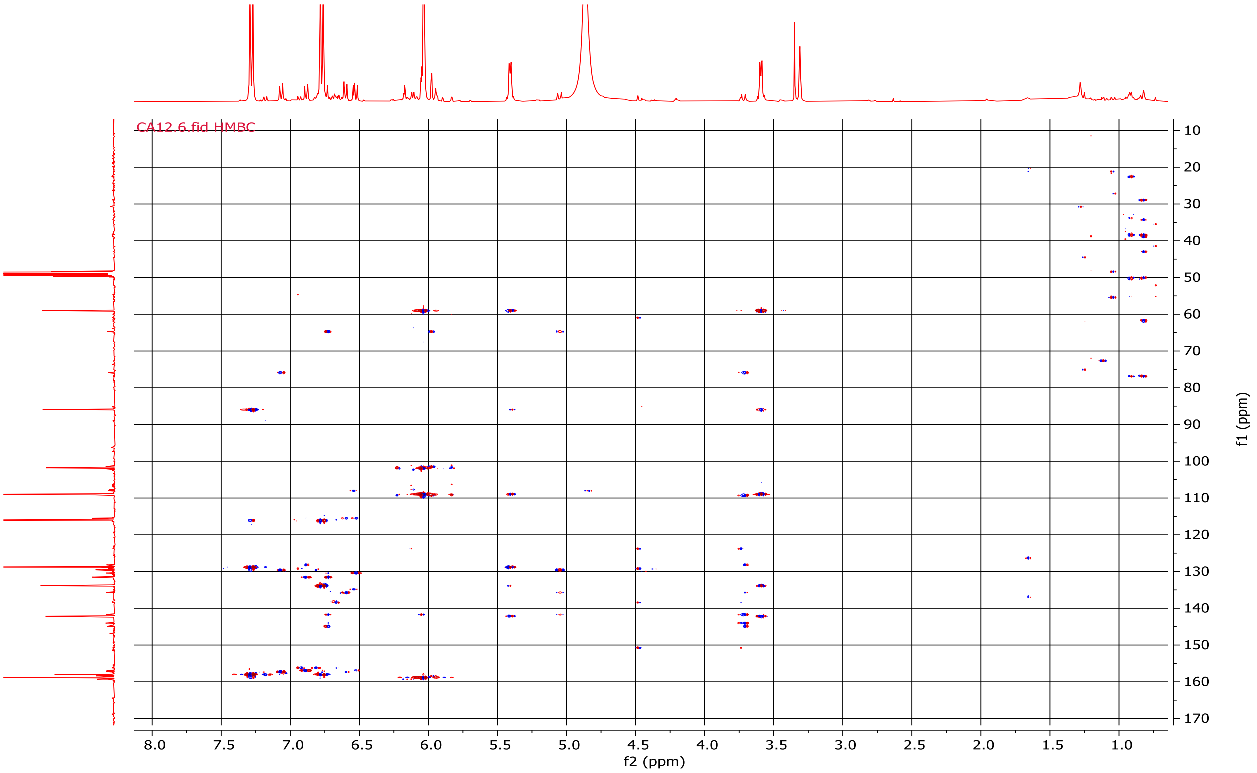

FIGURE S25: HMBC spectrum of compound **5**.

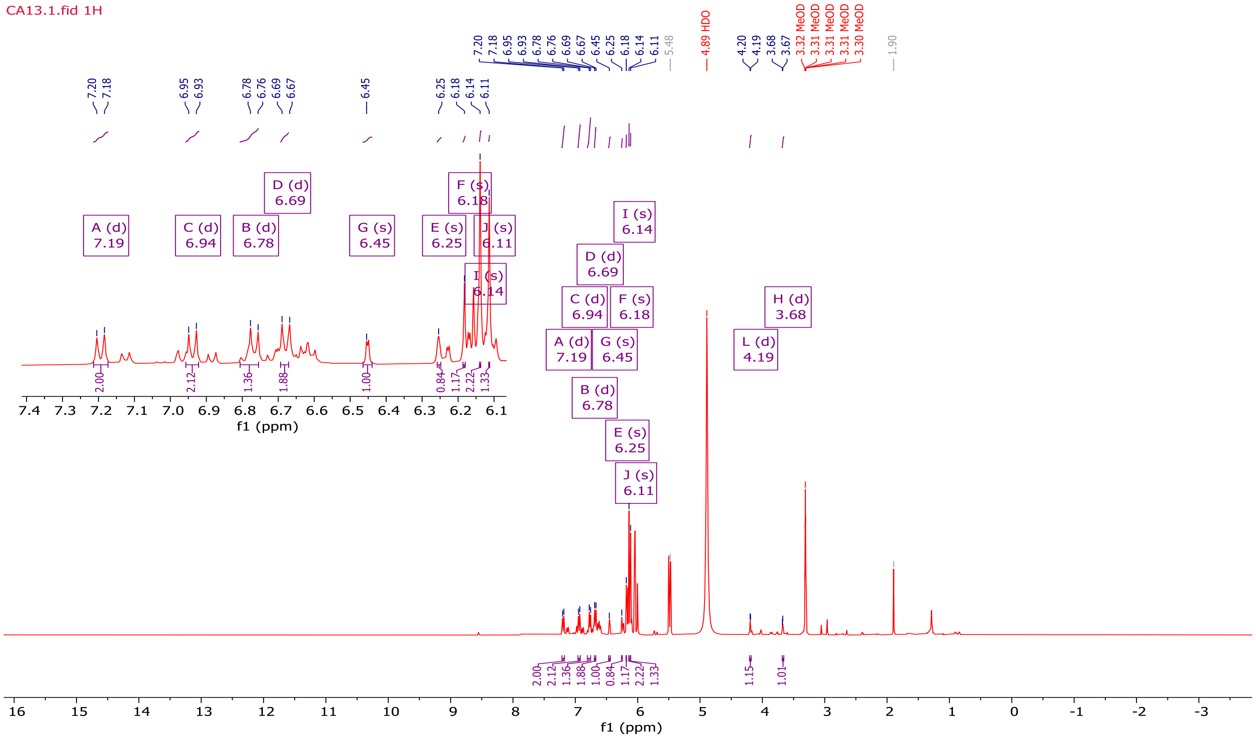

FIGURE S26: ^{1}H NMR spectrum of compound **6**.

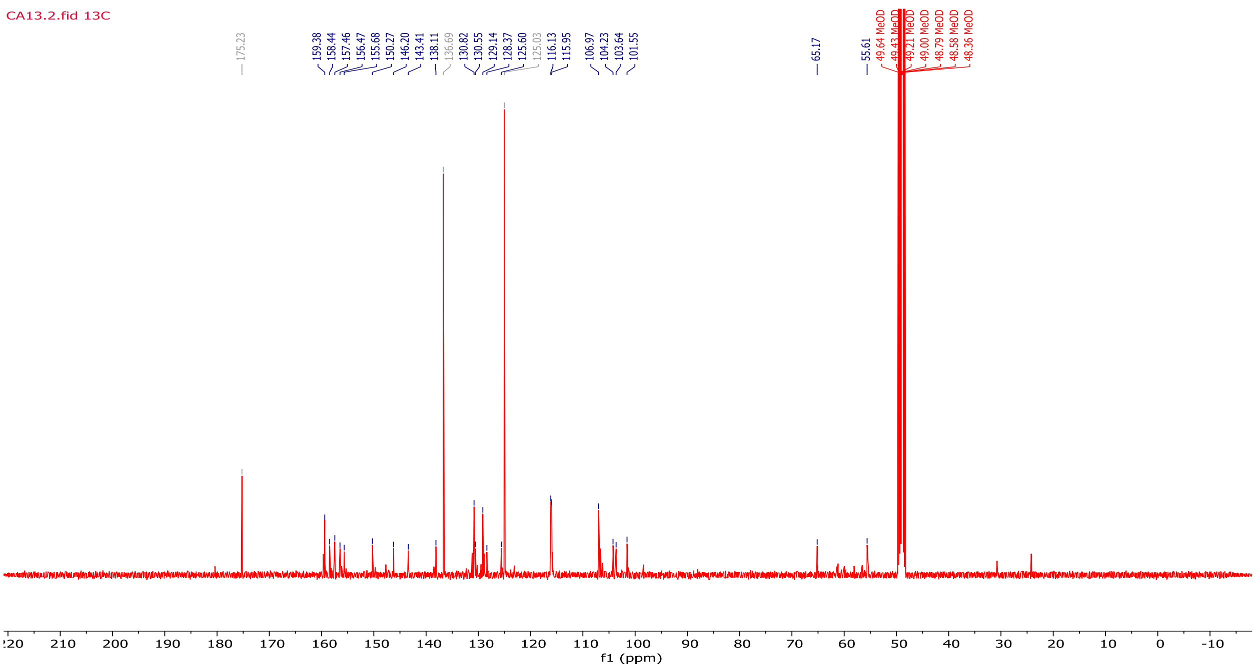

FIGURE S27: ^{13}C NMR spectrum of compound **6**.

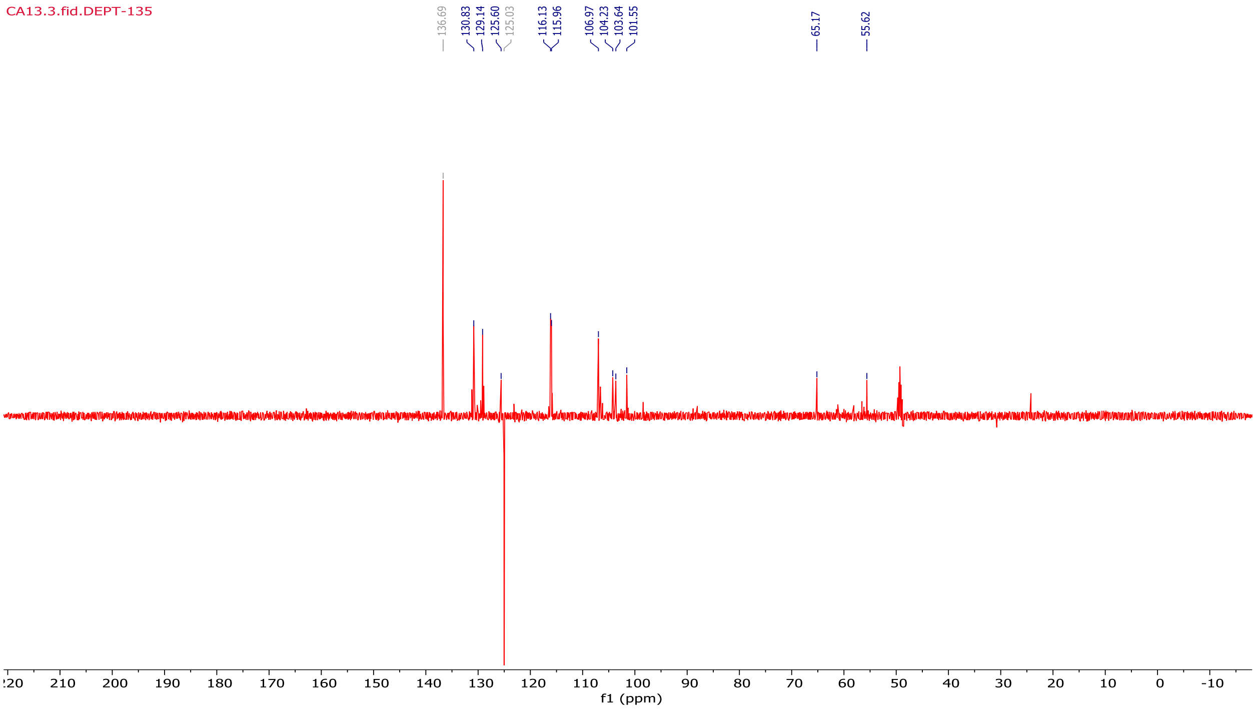

FIGURE S28: DEPT-135 spectrum of compound **6**.

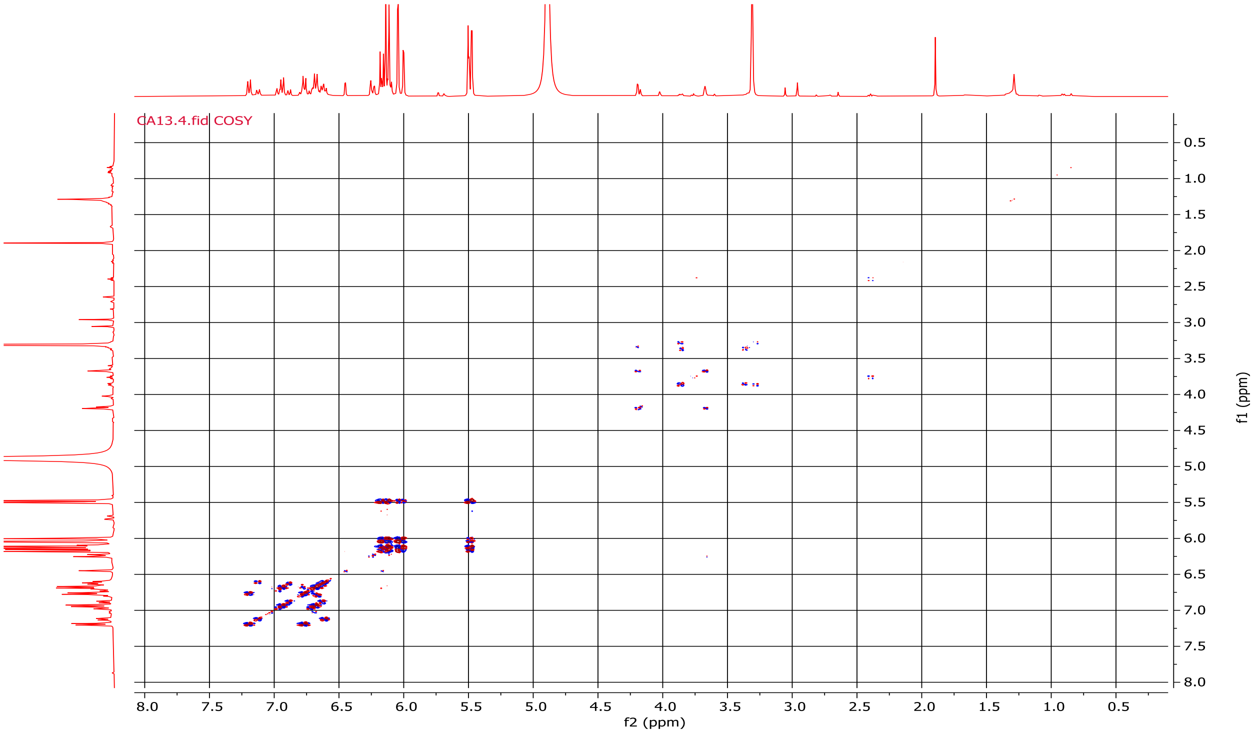

FIGURE S29: COSY spectrum of compound **6**.

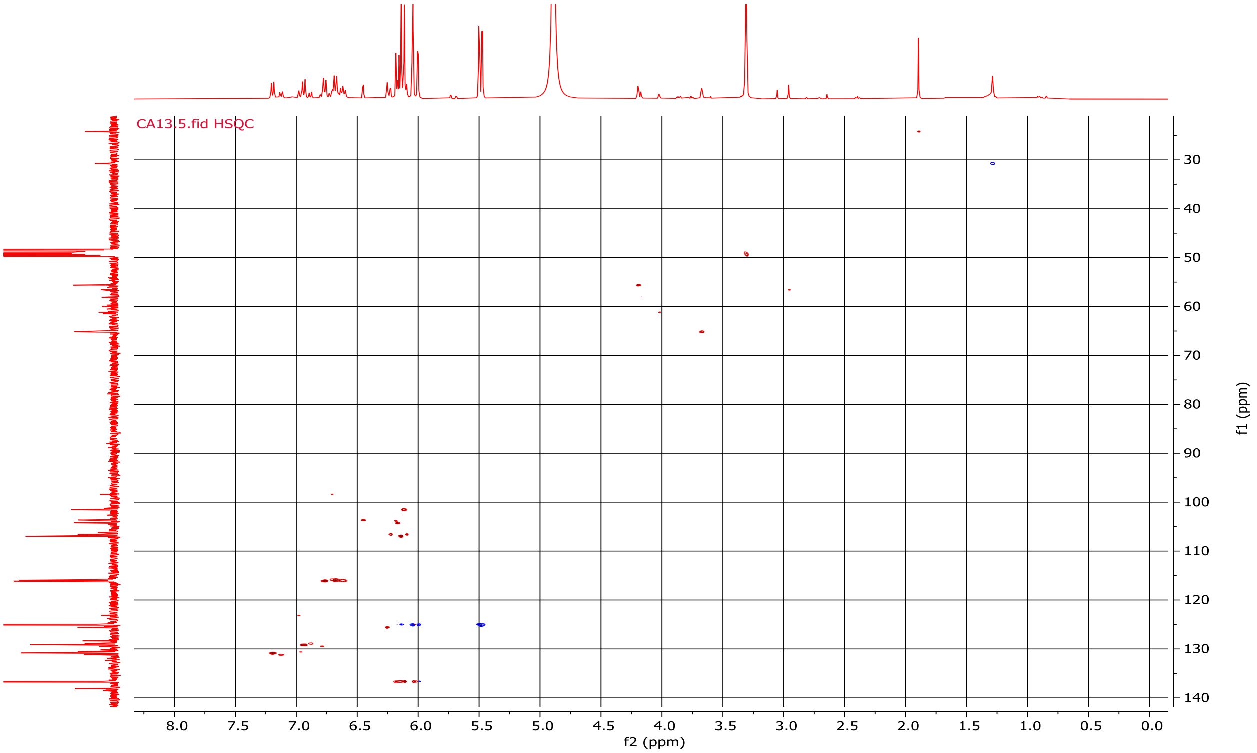

FIGURE S30: HSQC spectrum of compound **6**.

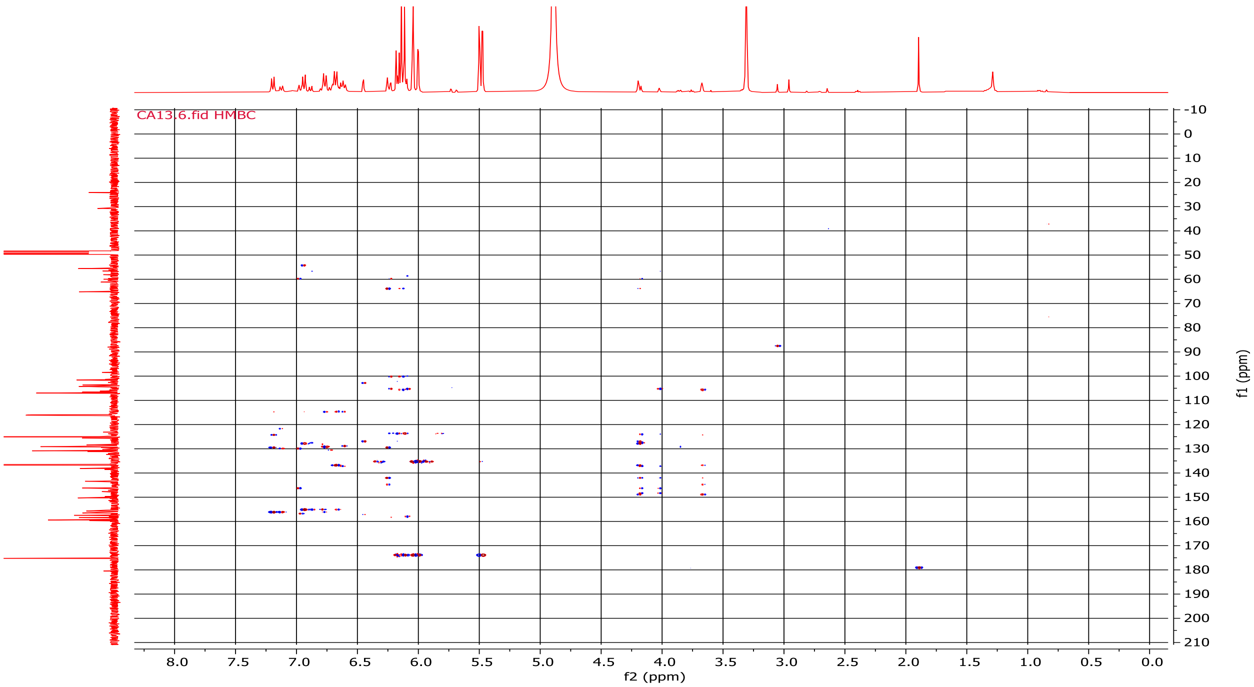

FIGURE S31: HMBC spectrum of compound **6**.
